# Supplementary material for: Association of functional health literacy and cognitive ability with self-reported diabetes in the English Longitudinal Study of Ageing: a prospective cohort study
Source: BMJ Open. 2022 Jun 3;12(6):e058496. doi: 10.1136/bmjopen-2021-058496 (PMC9171267; doi:10.1136/bmjopen-2021-058496)
Supplement: Supplementary data [file bmjopen-2021-058496supp001.pdf]

Supplementary material for:

The association of functional health literacy and cognitive ability with self-reported diabetes in the English Longitudinal Study of Ageing: A prospective cohort study

Fawns-Ritchie C, *et al. BMJ Open* 2022; 12:e058496. doi: 10.1136/bmjopen-2021-058496

Supplementary Table S1 Odds ratios (95% CI) from logistic regression models of the association between functional health literacy and cognitive ability with self-reported diabetes at wave 2

	Model 1: Health literacy n=8,293	Model 2: Cognitive ability n=8,335	Model 3: Health literacy and cognitive ability n=8,185	Model 4: +BMI and health behaviours n=6,302	Model 5: +CV comorbidities n=8,185	Model 6: +Education and social class n=7,861	Model 7: Fully-adjusted n=6,086
Health literacy							
Limited	Reference	-	Reference	Reference	Reference	Reference	Reference
Adequate	0.71*** (0.61, 0.84)		0.82* (0.69, 0.98)	0.97 (0.78, 1.21)	0.85 (0.72, 1.02)	0.84 (0.70, 1.01)	0.98 (0.78, 1.23)
Cognitive ability	-	0.73*** (0.67, 0.80)	0.78*** (0.70, 0.86)	0.90 (0.80, 1.02)	0.78*** (0.71, 0.87)	0.78*** (0.71, 0.87)	0.87 (0.76, 1.00)
Age	1.04*** (1.03, 1.05)	1.03*** (1.02, 1.04)	1.03*** (1.02, 1.04)	1.04*** (1.03, 1.06)	1.02*** (1.01, 1.03)	1.03*** (1.02, 1.04)	1.03*** (1.02, 1.05)
Age^2	0.998*** (0.997, 0.999)	0.998*** (0.997, 0.998)	0.998*** (0.997, 0.999)	0.998** (0.997, 0.999)	0.998*** (0.997, 0.999)	0.998*** (0.997, 0.999)	0.999 (0.998, 1.000)
Sex							
Female	Reference	Reference	Reference	Reference	Reference	Reference	Reference
Male	1.50*** (1.28, 1.77)	1.41*** (1.20, 1.66)	1.43 (1.22, 1.69)	2.16*** (1.75, 2.68)	1.45*** (1.23, 1.71)	1.44*** (1.22, 1.71)	2.09*** (1.67, 2.62)
BMI				1.10*** (1.08, 1.12)			1.09*** (1.07, 1.11)
Current smoking							
Non-smoker				Reference			Reference
Smoker				0.91 (0.66, 1.23)			0.93 (0.66, 1.27)
Alcohol consumption							
Daily/almost daily				Reference			Reference
At least once per week				1.21 (0.90, 1.65)			1.24 (0.91, 1.70)
At least once per month				1.78** (1.24, 2.56)			1.77** (1.21, 2.57)
Rarely				1.95*** (1.38, 2.76)			1.95*** (1.36, 2.79)
Never				2.40*** (1.67, 3.44)			2.12*** (1.45, 3.11)
Physical activity							
Inactive				Reference			Reference

Moderate activity	0.65*** (0.51, 0.83)			0.68** (0.53, 0.87)
Vigorous activity	0.50*** (0.37, 0.68)			0.56*** (0.41, 0.76)
Number of CV comorbidities		2.08*** (1.84, 2.36)		1.98*** (1.70, 2.32)
Number of CV comorbidities^2		0.88*** (0.84, 0.93)		0.88*** (0.82, 0.93)
Age left full-time education				
≤14 years			Reference	Reference
15-16 years			1.06 (0.84, 1.34)	1.16 (0.87, 1.56)
17-18 years			0.81 (0.56, 1.14)	0.98 (0.63, 1.50)
≥19 years			1.06 (0.74, 1.50)	1.32 (0.85, 2.05)
Social class				
Managerial and professional			Reference	Reference
Intermediate			0.79 (0.61, 1.02)	0.79 (0.58, 1.07)
Routine and manual			1.08 (0.87, 1.35)	1.01 (0.77, 1.32)

*p<.05, **p<.01, ***p<.001

Age^2, age squared; BMI, body mass index; CV, cardiovascular; number of CV cormorbidities^2, number of cardiovascular comorbidities squared.

Fawns-Ritchie C, *et al. BMJ Open* 2022; 12:e058496. doi: 10.1136/bmjopen-2021-058496

Supplementary Table S2 Hazard ratios (95% CI) from Cox regression models of the association between functional health literacy and cognitive ability with self-reporting diabetes during follow-up

	Model 1: Health literacy n=6,736 Events=490	Model 2: Cognitive ability n=6,746 Events=491	Model 3: Health literacy and cognitive ability n=6,654 Events=484	Model 4: +BMI health behaviours n=5,357 Events=377	Model 5: +CV comorbidities n=6654 Events=484	Model 6: +Education and social class n=6409 Events=462	Model 7: Fully-adjusted n=5,186 Events=360
Health literacy							
Limited	Reference	-	Reference	Reference	Reference	Reference	Reference
Adequate	0.64*** (0.53, 0.77)		0.72*** (0.59, 0.87)	0.79* (0.64, 0.99)	0.73** (0.60, 0.88)	0.79* (0.65, 0.97)	0.85 (0.68, 1.06)
Cognitive ability	-	0.77*** (0.69, 0.85)	0.79*** (0.71, 0.88)	0.85* (0.74, 0.96)	0.80*** (0.71, 0.89)	0.84** (0.75, 0.95)	0.88 (0.77, 1.01)
Age	1.01 (1.00, 1.02)	1.00 (0.99, 1.01)	1.00 (0.98, 1.01)	1.01 (1.00, 1.02)	0.99 (0.98, 1.00)	1.00 (0.98, 1.01)	1.01 (0.99, 1.02)
Sex							
Female	Reference	Reference	Reference	Reference	Reference	Reference	Reference
Male	1.43*** (1.20, 1.71)	1.39*** (1.16, 1.66)	1.38*** (1.15, 1.65)	1.84*** (1.49, 2.29)	1.38*** (1.16, 1.66)	1.39*** (1.15, 1.68)	1.82*** (1.45, 2.28)
BMI				1.12*** (1.10, 1.14)			1.12*** (1.10, 1.13)
Current smoking							
Non-smoker				Reference			Reference
Smoker				1.77*** (1.35, 2.31)			1.69*** (1.28, 2.22)
Alcohol consumption							
Daily/almost daily				Reference			Reference
At least once per week				1.11 (0.83, 1.49)			1.01 (0.75, 1.37)
At least once per month				1.53* (1.07, 2.19)			1.40 (0.97, 2.01)
Rarely				1.78*** (1.27, 2.50)			1.53* (1.08, 2.17)
Never				1.42 (0.95, 2.11)			1.15 (0.76, 1.73)
Physical activity							
Inactive				Reference			Reference
Moderate activity				0.78			0.79

	(0.61, 1.01)			(0.61, 1.03)
Vigorous activity	0.72* (0.54, 0.98)			0.76 (0.56, 1.04)
Number of CV comorbidities		1.34*** (1.22, 1.46)		1.17** (1.05, 1.30)
Age left full-time education				
≤14 years			Reference	Reference
15-16 years			0.93 (0.71, 1.22)	1.00 (0.74, 1.36)
17-18 years			0.61* (0.41, 0.91)	0.73 (0.47, 1.15)
≥19 years			0.44*** (0.28, 0.68)	0.58* (0.35, 0.96)
Social class				
Managerial and professional			Reference	Reference
Intermediate			0.81 (0.62, 1.07)	0.91 (0.66, 1.24)
Routine and manual			1.17 (0.93, 1.49)	1.17 (0.89, 1.53)

**p*<.05, ***p*<.01, ****p*<.001
BMI, body mass index; CV, cardiovascular.

Supplementary Table S3 Odds ratios (95% CI) for the association between functional health literacy and cognitive ability with cross-sectional diabetes status at wave 2 in a sub-sample of 6,086 participants with data on all variables of interest

	Model 1: Health literacy	Model 2: Cognitive ability	Model 3: Health literacy and cognitive ability	Model 4: +BMI and health behaviours	Model 5: +CV comorbidities	Model 6: +Education and social class	Model 7: Fully-adjusted
Health literacy							
Limited	Reference	-	Reference	Reference	Reference	Reference	Reference
Adequate	0.79* (0.64, 0.97)		0.88 (0.71, 1.10)	0.96 (0.77, 1.20)	0.92 (0.74, 1.15)	0.90 (0.72, 1.12)	0.98 (0.78, 1.23)
Cognitive ability	-	0.78*** (0.69, 0.88)	0.79*** (0.70, 0.90)	0.88 (0.77, 1.00)	0.80*** (0.70, 0.91)	0.82** (0.72, 0.93)	0.88 (0.77, 1.00)
Age	1.04*** (1.03, 1.06)	1.03*** (1.02, 1.05)	1.03*** (1.02, 1.05)	1.04*** (1.02, 1.05)	1.02** (1.01, to 1.04)	1.04*** (1.02, 1.05)	1.03*** (1.02, 1.05)
Age^2	0.999* (0.997, 1.000)	0.999** (0.997, 1.000)	0.999** (0.997, 1.000)	0.998** (0.997, 1.000)	0.999 (0.998, 1.000)	0.999* (0.997, 1.000)	0.999 (0.998, 1.000)
Sex							
Female	Reference	Reference	Reference	Reference	Reference	Reference	Reference
Male	1.66*** (1.36, 2.03)	1.58*** (1.29, 1.93)	1.58*** (1.29, 1.94)	2.17*** (1.74, 2.70)	1.63*** (1.33, 2.00)	1.56 (1.27, 1.92)	2.09*** (1.67, 2.62)
BMI				1.10*** (1.08, 1.12)			1.09*** (1.07, 1.11)
Current smoking							
Non-smoker				Reference			Reference
Smoker				0.89 (0.64, 1.22)			0.93 (0.66, 1.27)
Alcohol consumption							
Daily/almost daily				Reference			Reference
At least once per week				1.21 (0.90, 1.66)			1.24 (0.91, 1.70)
At least once per month				1.76** (1.21, 2.54)			1.77** (1.21, 2.57)
Rarely				2.01*** (1.42, 2.87)			1.95*** (1.36, 2.79)
Never				2.24*** (1.55, 3.26)			2.12*** (1.45, 3.11)
Physical activity							
Inactive				Reference			Reference
Moderate activity				0.65***			0.68**

	(0.51, 0.82)			(0.53, 0.87)
Vigorous activity	0.51*** (0.37, 0.69)			0.56*** (0.41, 0.76)
Number of CV comorbidities		2.22*** (1.91, 2.59)		1.98*** (1.70, 2.32)
Number of CV comorbidities^2		0.87*** (0.81, 0.92)		0.88*** (0.82, 0.93)
Education				
≤14 years			Reference	Reference
15-16 years			1.07 (0.81, 1.42)	1.17 (0.87, 1.56)
17-18 years			0.78 (0.51, 1.18)	0.98 (0.64, 1.50)
≥19 years			0.94 (0.62, 1.43)	1.32 (0.85, 2.05)
Social class				
Managerial and professional			Reference	Reference
Intermediate			0.79 (0.59, 1.07)	0.79 (0.58, 1.07)
Routine and manual			1.09 (0.84, 1.42)	1.01 (0.77, 1.32)

**p*<.05, ***p*<.01, ****p*<.001

Age^2, age squared; BMI, body mass index; CV, cardiovascular; number of CV cormorbidities^2, number of cardiovascular comorbidities squared.

Supplementary Table S4 Hazard ratios (95% CI) from Cox regression models of the association between functional health literacy and cognitive ability with self-reporting diabetes during follow-up. Models are run on a sub-sample of 5,186 (360 with diabetes) participants with data on all variables of interest

	Model 1: Health literacy	Model 2: Cognitive ability	Model 3: Health literacy and cognitive ability	Model 4: +BMI and health behaviours	Model 5: +CV comorbidities	Model 6: +Education and social class	Model 7: Fully-adjusted
Health literacy							
Limited	Reference	-	Reference	Reference	Reference	Reference	Reference
Adequate	0.64*** (0.52, 0.80)		0.73** (0.58, 0.91)	0.80 (0.64, 1.01)	0.74** (0.59, 0.93)	0.79* (0.63, 0.98)	0.85 (0.68, 1.06)
Cognitive ability	-	0.72*** (0.63, 0.82)	0.76*** (0.66, 0.86)	0.84** (0.73, 0.96)	0.76*** (0.67, 0.87)	0.83** (0.72, 0.95)	0.88 (0.77, 1.01)
Age	1.01 (0.997, 1.02)	1.00 (0.98, 1.01)	1.00 (0.98, 1.01)	1.01 (0.997, 1.03)	0.99 (0.98, 1.01)	1.00 (0.98, 1.01)	1.01 (0.99, 1.02)
Sex							
Female	Reference	Reference	Reference	Reference	Reference	Reference	Reference
Male	1.47*** (1.20, 1.81)	1.38** (1.12, 1.70)	1.40** (1.13, 1.72)	1.82*** (1.46, 2.27)	1.40** (1.14, 1.73)	1.42** (1.15, 1.76)	1.82*** (1.45, 2.28)
BMI				1.12*** (1.10, 1.14)			1.12*** (1.10, 1.13)
Current smoking							
Non-smoker				Reference			Reference
Smoker				1.79*** (1.36, 2.34)			1.69*** (1.28, 2.22)
Alcohol consumption							
Daily/almost daily				Reference			Reference
At least once per week				1.10 (0.80, 1.46)			1.01 (0.75, 1.36)
At least once per month				1.49* (1.03, 2.14)			1.40 (0.97, 2.01)
Rarely				1.70** (1.20, 2.40)			1.53* (1.08, 2.17)
Never				1.30 (0.86, 1.96)			1.15 (0.76, 1.73)
Physical activity							
Inactive				Reference			Reference
Moderate activity				0.76* (0.59, 0.99)			0.79 (0.61, 1.03)
Vigorous activity				0.71*			0.76

	(0.52, 0.96)			(0.56, 1.04)
Number of CV comorbidities		1.30*** (1.17, 1.45)		1.17** (1.05, 1.30)
Education				
≤14 years			Reference	Reference
15-16 years			0.90 (0.67, 1.23)	1.00 (0.74, 1.36)
17-18 years			0.56* (0.36, 0.88)	0.73 (0.47, 1.15)
≤19 years			0.44** (0.27, 0.73)	0.58* (0.35, 0.96)
Social class				
Managerial and professional			Reference	Reference
Intermediate			0.86 (0.63, 1.17)	0.91 (0.66, 1.24)
Routine and manual			1.24 (0.95, 1.63)	1.16 (0.89, 1.53)

*p<.05, **p<.01, ***p<.001
BMI, body mass index; CV, cardiovascular.

Fawns-Ritchie C, *et al. BMJ Open* 2022; 12:e058496. doi: 10.1136/bmjopen-2021-058496

Supplementary Table S5 Odds ratios (95% CI) from logistic regression models of the association between functional health literacy and cognitive ability with self-reported diabetes at wave 2. Models are run on a sub-sample of participants with HbA_{1c} levels recorded wave 2, removing participants with suspected undiagnosed diabetes (n=5,671; 399 with diabetes)

	Model 1: Health literacy n= 5533	Model 2: Cognitive ability n=5534	Model 3: Health literacy and cognitive ability n=5470	Model 4: +BMI and health behaviours n=4845	Model 5: +CV comorbidities n=5470	Model 6: +Education and social class n=5271	Model 7: Fully-adjusted n=4674
Health literacy							
Limited	Reference	-	Reference	Reference	Reference	Reference	Reference
Adequate	0.91 (0.73, 1.14)		1.04 (0.82, 1.32)	1.17 (0.90, 1.53)	1.07 (0.84, 1.36)	1.04 (0.82, 1.33)	1.14 (0.87, 1.51)
Cognitive ability	-	0.78*** (0.69, 0.88)	0.78*** (0.69, 0.89)	0.88 (0.76, 1.03)	0.78*** (0.69, 0.89)	0.80** (0.69, 0.92)	0.85 (0.73, 1.01)
Age	1.04*** (1.03, 1.05)	1.03*** (1.02, 1.05)	1.03*** (1.01, 1.04)	1.04*** (1.02, 1.05)	1.02* (1.00, 1.03)	1.03*** (1.01, 1.04)	1.03** (1.01, 1.05)
Age^2	0.998*** (0.996, 0.999)	0.997*** (0.996, 0.999)	0.998*** (0.996, 0.999)	0.998** (0.996, 0.999)	0.998** (0.997, 0.999)	0.998** (0.996, 0.999)	0.999 (0.997, 1.000)
Sex							
Female	Reference	Reference	Reference	Reference	Reference	Reference	Reference
Male	1.45*** (1.17, 1.79)	1.39** (1.13, 1.72)	1.38** (1.11, 1.71)	2.16*** (1.67, 2.79)	1.40** (1.13, 1.74)	1.38** (1.10, 1.72)	2.09*** (1.60, 2.74)
BMI				1.11*** (1.09, 1.14)			1.10*** (1.07, 1.12)
Current smoking							
Non-smoker				Reference			Reference
Smoker				0.83 (0.56, 1.20)			0.86 (0.57, 1.26)
Alcohol consumption							
Daily/almost daily				Reference			Reference
At least once per week				1.33 (0.94, 1.92)			1.42 (0.98, 2.08)
At least once per month				1.87** (1.22, 2.87)			1.95** (1.25, 3.07)
Rarely				2.08*** (1.38, 3.16)			2.22*** (1.44, 3.44)
Never				2.23*** (1.43, 3.49)			1.85* (1.14, 3.00)

Physical activity				
Inactive	Reference			Reference
Moderate activity	0.65** (0.49, 0.87)			0.68* (0.50, 0.92)
Vigorous activity	0.40*** (0.27, 0.57)			0.43*** (0.29, 0.63)
Number of CV comorbidities		2.30*** (1.96, 2.70)		2.08*** (1.73, 2.50)
Number of CV comorbidities^2		0.88*** (0.82, 0.94)		0.90** (0.82, 0.97)
Age left full-time education				
≤14 years			Reference	Reference
15-16 years			1.00 (0.74, 1.37)	1.31 (0.92, 1.87)
17-18 years			0.63 (0.39, 1.00)	0.94 (0.55, 1.58)
≥19 years			0.84 (0.53, 1.32)	1.23 (0.72, 2.08)
Social class				
Managerial and professional			Reference	Reference
Intermediate			0.64** (0.46, 0.89)	0.62* (0.42, 0.89)
Routine and manual			0.94 (0.71, 1.24)	0.89 (0.65, 1.22)

*p<.05, **p<.01, ***p<.001

Age^2, age squared; BMI, body mass index; CV, cardiovascular; number of CV cormorbidities^2, number of cardiovascular comorbidities squared.

Supplementary Table S6 Hazard ratios (95% CI) from Cox regression models of the association between functional health literacy and cognitive ability with reporting diabetes during follow-up. Models are run on a sub-sample of participants with HbA_{1c} levels recorded at wave 4 and/or wave 6, removing participants with suspected undiagnosed diabetes (n=4,206; 212 with diabetes)

	Model 1: Health literacy n=3675 Events=203	Model 2: Cognitive ability n=3674 Events=205	Model 3: Health literacy and cognitive ability n=3641 Events=201	Model 4: +BMI health behaviours n=3184 Events=165	Model 5: +CV comorbidities n=3641 Events=201	Model 6: +Education and social class n=3530 Events=195	Model 7: Fully-adjusted n=3095 Events=159
Health literacy							
Limited	Reference	-	Reference	Reference	Reference	Reference	Reference
Adequate	0.50*** (0.38, 0.67)		0.58*** (0.43, 0.77)	0.63** (0.45, 0.88)	0.59*** (0.44, 0.79)	0.61*** (0.45, 0.82)	0.65* (0.46, 0.90)
Cognitive ability	-	0.71*** (0.60, 0.84)	0.78** (0.65, 0.94)	0.91 (0.74, 1.11)	0.78** (0.66, 0.94)	0.83* (0.68, 0.997)	0.93 (0.76, 1.16)
Age	1.02* (1.00, 1.03)	1.01 (0.99, 1.03)	1.01 (0.99, 1.03)	1.02* (1.00, 1.04)	1.00 (0.98, 1.02)	1.01 0.99, 1.03)	1.01 (0.99, 1.04)
Sex							
Female	Reference	Reference	Reference	Reference	Reference	Reference	Reference
Male	1.57** (1.19, 2.06)	1.50** (1.14, 1.98)	1.51** (1.14, 2.00)	2.22 *** (1.59, 3.10)	1.53** (1.15, 2.02)	1.50** (1.12, 2.00)	2.11*** (1.49, 2.97)
BMI				1.12*** (1.10, 1.15)			1.11*** (1.08, 1.14)
Current smoking							
Non-smoker				Reference			Reference
Smoker				2.12*** (1.43, 3.15)			2.04*** (1.36, 3.06)
Alcohol consumption							
Daily/almost daily				Reference			Reference
At least once per week				1.09 (0.70, 1.71)			1.00 (0.64, 1.57)
At least once per month				1.48 (0.86, 2.55)			1.34 (0.77, 2.34)
Rarely				2.10*** (1.27, 3.48)			1.78* (1.06, 2.98)
Never				1.54 (0.85, 2.80)			1.27 (0.69, 2.35)
Physical activity							
Inactive				Reference			Reference

Moderate activity	0.64* (0.43, 0.94)			0.65* (0.44, 0.96)
Vigorous activity	0.61* (0.39, 0.95)			0.65 (0.41, 1.02)
Number of CV comorbidities		1.43*** (1.25, 1.64)		1.22* (1.04, 1.44)
Age left full-time education				
≤14 years			Reference	Reference
15-16 years			1.01 (0.66, 1.54)	0.91 (0.57, 1.44)
17-18 years			0.71 (0.39, 1.29)	0.78 (0.41, 1.48)
≥19 years			0.52 (0.27, 1.02)	0.59 (0.28, 1.23)
Social class				
Managerial and professional			Reference	Reference
Intermediate			0.83 (0.54, 1.27)	0.84 (0.52, 1.35)
Routine and manual			1.22 (0.85, 1.74)	1.20 (0.80, 1.79)

$*p<.05$, $**p<.01$, $***p<.001$

BMI, body mass index; CV, cardiovascular.

Fawns-Ritchie C, *et al. BMJ Open* 2022; 12:e058496. doi: 10.1136/bmjopen-2021-058496
