# Supplementary material for: Association of functional health literacy and cognitive ability with self-reported diabetes in the English Longitudinal Study of Ageing: a prospective cohort study
Source: BMJ Open. 2022 Jun 3;12(6):e058496. doi: 10.1136/bmjopen-2021-058496 (PMC9171267; doi:10.1136/bmjopen-2021-058496)
Supplement: Author's manuscript [file bmjopen-2021-058496.draft_revisions.pdf]

BMJ Open

BMJ Open is committed to open peer review. As part of this commitment we make the peer review history of every article we publish publicly available.

When an article is published we post the peer reviewers' comments and the authors' responses online. We also post the versions of the paper that were used during peer review. These are the versions that the peer review comments apply to.

The versions of the paper that follow are the versions that were submitted during the peer review process. They are not the versions of record or the final published versions. They should not be cited or distributed as the published version of this manuscript.

BMJ Open is an open access journal and the full, final, typeset and author-corrected version of record of the manuscript is available on our site with no access controls, subscription charges or pay-per-view fees (http://bmjopen.bmj.com).

If you have any questions on BMJ Open's open peer review process please email info.bmjopen@bmj.com

BMJ Open

The association of health literacy and cognitive ability with self-reported diabetes in the English Longitudinal Study of Ageing

Journal:	*BMJ Open*
Manuscript ID	bmjopen-2021-058496
Article Type:	Original research
Date Submitted by the Author:	20-Oct-2021
Complete List of Authors:	Fawns-Ritchie, Chloe; The University of Edinburgh, Department of Psychology<br>Price, Jackie; The University of Edinburgh, Usher Institute<br>Deary, Ian; The University of Edinburgh, Department of Psychology
Keywords:	General diabetes < DIABETES & ENDOCRINOLOGY, EPIDEMIOLOGY, Diabetes & endocrinology < INTERNAL MEDICINE, PUBLIC HEALTH

SCHOLARONE™
Manuscripts

BMJ

I, the Submitting Author has the right to grant and does grant on behalf of all authors of the Work (as defined in the below author licence), an exclusive licence and/or a non-exclusive licence for contributions from authors who are: i) UK Crown employees; ii) where BMJ has agreed a CC-BY licence shall apply, and/or iii) in accordance with the terms applicable for US Federal Government officers or employees acting as part of their official duties; on a worldwide, perpetual, irrevocable, royalty-free basis to BMJ Publishing Group Ltd ("BMJ") its licensees and where the relevant Journal is co-owned by BMJ to the co-owners of the Journal, to publish the Work in this journal and any other BMJ products and to exploit all rights, as set out in our licence.

The Submitting Author accepts and understands that any supply made under these terms is made by BMJ to the Submitting Author unless you are acting as an employee on behalf of your employer or a postgraduate student of an affiliated institution which is paying any applicable article publishing charge ("APC") for Open Access articles. Where the Submitting Author wishes to make the Work available on an Open Access basis (and intends to pay the relevant APC), the terms of reuse of such Open Access shall be governed by a Creative Commons licence – details of these licences and which Creative Commons licence will apply to this Work are set out in our licence referred to above.

Other than as permitted in any relevant BMJ Author's Self Archiving Policies, I confirm this Work has not been accepted for publication elsewhere, is not being considered for publication elsewhere and does not duplicate material already published. I confirm all authors consent to publication of this Work and authorise the granting of this licence.

For peer review only

The association of health literacy and cognitive ability with self-reported diabetes in the English Longitudinal Study of Ageing

Chloe Fawns-Ritchie[1,2], Jackie Price[3], and Ian J Deary[1,2]

[1]Department of Psychology, University of Edinburgh, Edinburgh, UK

[2]Centre for Cognitive Ageing and Cognitive Epidemiology, University of Edinburgh, Edinburgh, UK

[3]Usher Institute, University of Edinburgh, Edinburgh, UK

Corresponding author:

Chloe Fawns-Ritchie

Department of Psychology, University of Edinburgh

7 George Square

Edinburgh, EH8 9JZ

Scotland, UK

Telephone: +44 131 650 8317

c.fawns-ritchie@ed.ac.uk

Abstract word count: 247

Manuscript word count: 4316

For peer review only

ABSTRACT

Background Lower health literacy and cognitive ability – variables which are moderately correlated – have associations with greater diabetes risk. This study investigated whether health literacy and cognitive ability were associated with diabetes risk when examined individually and simultaneously.

Method Participants were 8,669 English Longitudinal Study of Ageing participants (mean age=66.7, SD=9.7) who completed health literacy and cognitive ability tests at wave 2 (2004-2005), and who answered a self-reported question on whether a doctor had ever diagnosed them with diabetes. Logistic regression was used to examine cross-sectional associations of health literacy and cognitive ability with diabetes status. Cox regression was used to test the associations of health literacy and cognitive ability with risk of diabetes over a median of 9.5 years follow-up.

Results Adequate (compared to limited) health literacy (OR=0.72, 95% CI=0.61 to 0.84) and higher cognitive ability (OR per 1 SD=0.73, 95% CI=0.67 to 0.80) were associated with lower odds of diabetes. Adequate health literacy (HR=0.64; 95% CI=0.53 to 0.77) and higher cognitive ability (HR=0.77, 95% CI=0.69 to 0.85) were associated with lower risk of diabetes during follow-up. When both health literacy and cognitive ability were added to the same model, these associations were slightly attenuated. Additionally adjusting for health behaviours and BMI fully attenuated cross-sectional associations between health literacy and cognitive ability with diabetes status, and partly attenuated associations between health literacy and cognitive ability with risk of diabetes during follow-up.

Conclusions Adequate health literacy and better cognitive ability were independently associated with reduced risk of diabetes.

For peer review only - http://bmjopen.bmj.com/site/about/guidelines.xhtml

Keywords Health literacy, cognition, diabetes risk, prospective studies

For peer review only

For peer review only - http://bmjopen.bmj.com/site/about/guidelines.xhtml

Strengths and limitations of this study

- This study used data from the English Longitudinal Study of Ageing, a large prospective cohort study designed to be representative of community-dwelling adults aged over 50 years living in England.
- Participants were followed-up for a median of 9.5 years to determine whether they were diagnosed with diabetes.
- Diabetes status was self-reported.

For peer review only

For peer review only - http://bmjopen.bmj.com/site/about/guidelines.xhtml

INTRODUCTION

Diabetes is a common chronic condition in older adulthood and is associated with substantial morbidity and mortality.[1] Type 2 diabetes, the most common type of diabetes, is at least partly preventable.[1] Understanding the characteristics of those most at risk of developing diabetes is important for appropriately targeting diabetes education and interventions. Risk factors for developing diabetes include older age, deprivation, and obesity.[1]

Lower cognitive ability may be a risk factor for diabetes. Whereas one study[2] found that childhood cognitive ability did not predict diabetes in midlife, others have found that lower cognitive ability in early life was associated with higher risk of diabetes in adulthood.[3, 4] In a sample of Scottish older adults who had their cognitive ability tested in childhood,[3] a 1 SD advantage in cognitive ability was associated with 26% lower odds of reporting diabetes in older age. Individuals with higher cognitive ability might have the cognitive skills required to self-manage their health, take better care of themselves throughout life, and thus reduce the risk of developing diabetes.[3, 5]

Health literacy – the "capacity to obtain, process and understand basic health information and services needed to make basic health decisions"[6] – might also play a role in diabetes risk. In cross-sectional studies, rates of diabetes are higher in those with low health literacy.[7, 8] In one study, participants with inadequate health literacy were 48% more likely to report having diabetes when compared to participants with adequate health literacy after adjusting for sociodemographic and health variables.[8] Individuals with lower health literacy might lack the health-related skills required to obtain, understand and follow health advice, such as eating well and exercising, which might reduce the risk of diabetes.[6]

In patients with diabetes, higher health literacy has consistently been associated with greater diabetes knowledge.[9-11] A very small association between higher health literacy and lower

For peer review only - http://bmjopen.bmj.com/site/about/guidelines.xhtml

HbA_{1C} levels in patients with diabetes has been reported in a meta-analysis of 26 studies (r=-0.048, p=0.027).[10] Whereas studies have investigated the association between health literacy and disease management in people with diabetes, little is known about whether health literacy is associated with risk of developing diabetes.

Health literacy and cognitive ability test scores are positively correlated.[12, 13] Rank-order correlations between general cognitive ability and three health literacy tests ranged from 0.37 to 0.50.[13] Researchers have sought to determine the role of cognitive ability in the association between health literacy and a range of health outcomes. Most (but not all)[14] studies have found that cognitive ability partly or entirely attenuates the association between health literacy and health.[15-18] Any association between health literacy and diabetes may be attenuated when also measuring cognitive ability.

The aim of the current study was to better understand the associations of health literacy and cognitive ability with risk of diabetes. Using data from the English Longitudinal Study of Ageing (ELSA), a cohort study designed to be representative of adults aged 50 years and over living in England,[19] the present study investigated whether health literacy and cognitive ability were independently associated with self-reported diabetes status. First, the cross-sectional associations between health literacy, cognitive ability, and self-reported diabetes were investigated. Second, participants without diabetes at baseline were followed-up for up to 10 years to determine whether health literacy and cognitive ability were independently associated with risk of diabetes in mid-to-later life.

METHODS

Participants

This study used data from core members of the ELSA study, a prospective cohort study of community-dwelling adults residing in England. The wave 1 (2002-2003) sample consisted of

For peer review only - http://bmjopen.bmj.com/site/about/guidelines.xhtml

11,391 participants who had previously participated in the Health Survey for England and who were living in a private household.[19] ELSA participants have been followed up every two years.

A face-to-face interview was used to measure topics including health, lifestyle and economic circumstances. Participants answered a self-completion questionnaire including questions about diet and alcohol consumption. A nurse visit was carried out every second wave to assess physical measurements including height and weight, and blood and saliva samples were taken to measure biomarkers of disease. Detailed descriptions of the sample design and data collected in ELSA are reported elsewhere.[19] The present study used data from waves 2 to 7, and baseline, here, was considered to be Wave 2 (2004-2005; n=8,726), which was when the health literacy assessment was introduced.

Ethical approval was obtained from the NHS Multicentre Research Ethics Committee, London (reference: MREC/01/2/91). Written informed consent was obtained from all ELSA participants. This study confirmed to the principles embodied in the Declaration of Helsinki.

Patient and Public Involvement

Participants were not involved in the development of any part of this study.

Measures

Diabetes

Two measures of diabetes were used as outcome variables.

Baseline diabetes status: Individuals who answered “yes” to “Has a doctor ever told you that you have diabetes?” at wave 2 were categorised as having diabetes. This question did not differentiate which type of diabetes the participant was diagnosed with.

For peer review only - http://bmjopen.bmj.com/site/about/guidelines.xhtml

Incident diabetes: For incident diabetes, the analysis was restricted to participants who did not self-report diabetes at wave 2 and who had at least one wave of follow-up between waves 3 and 7. Participants who did not self-report diabetes at wave 2 and who subsequently answered "yes" to "Has a doctor ever told you that you have diabetes?" any time between waves 3 and 7 were categorised as having incident diabetes. As all participants were aged over 50 years at diagnosis, these cases are probably cases of type 2 diabetes.

Date of diabetes diagnosis: Individuals who self-reported diabetes were asked which month and year they were diagnosed. Date of diabetes diagnosis was used to calculate the time between wave 2 assessment and diabetes diagnosis.

Health literacy

A brief 4-item health literacy test was administered during the interview at wave 2. This test assessed health-related reading comprehension skills which are thought to be required to successfully understand written health materials commonly encountered in healthcare. Participants were presented with a piece of paper containing a label for a packet of over-the-counter medication. Participants were asked four questions about the information on this label (e.g., "what is the maximum number of days you may take this medicine?"). The score was the number of correctly answered questions. As has been done in other studies,[20, 21] performance was categorised as adequate (4/4 correct) or limited (<4 correct).

Cognitive ability

Four tests administered during the wave 2 interview were used to measure general cognitive ability. Immediate and delayed word recall tests were used to assess verbal declarative memory. For immediate and delayed recall, participants were read a list of 10 words and were asked to immediately recall as many of the words as possible. The score was the number of words recalled immediately. After a short delay, in which the words were not repeated,

For peer review only - http://bmjopen.bmj.com/site/about/guidelines.xhtml

participants were asked to remember the 10 words again. The score was the number of words recalled after a delay. Executive function was assessed with verbal fluency. Participants were instructed to name as many animals as possible. The score was the number of animals named within 60 seconds. Letter cancellation was used to assess processing speed. Participants were presented with a piece of paper containing letters of the alphabet arranged in rows and columns. The task was to scan the piece of paper and score out all Ps and Ws. The score was the combined number of Ps and Ws scored out in 60 seconds.

Scores of 0 on animal fluency (n=48) and letter cancellation (n=3) were removed as this suggests participants did not complete the task or did not understand the task. Scores of ≥50 on animal fluency (n=4), and ≥60 on the letter cancellation (n=3) were removed as these scores were questionably high given the time limits. Scores on the four cognitive ability tests were entered into a principal component analysis (PCA). Only the first component had an eigenvalue >1. The scree plot also indicated one component. Scores from the first principal component were saved and used as a measure of cognitive ability (mean=0.00, SD=1.00). The first component accounted for 57% of the variance in the scores on the four cognitive tests. The loadings were: Immediate word recall=0.83, delayed word recall=0.85, animal fluency=0.72, and letter cancellation=0.58.

Covariates

Age (in years), sex, BMI, health behaviours, number of cardiovascular comorbidities, and measures of socioeconomic status were used as covariates. Unless otherwise stated, all were self-reported at the wave 2 interview. Participants aged over 90 years had their age set to 90 as there were so few of them. Participants were asked whether they smoked cigarettes nowadays and were categorised as current smokers or non-smokers. Participants were asked how often they took part in moderate and vigorous physical activity (more than once a week, once a

For peer review only - http://bmjopen.bmj.com/site/about/guidelines.xhtml

For peer review only

week, one to three times a month, and hardly ever/never). Physical activity levels were categorised as vigorous activity at least once per week, moderate activity at least once per week, and physically inactive. Participants were asked about their frequency of alcohol consumption in the past 12 months in the self-completion questionnaire. This was categorised as never, rarely, at least once a month, at least once a week, and daily/almost daily. Height and weight, measured during the wave 2 nurse interview, were used to calculate BMI (kg/m^2). Cardiovascular comorbidities were assessed by counting the number of self-reported cardiovascular conditions from hypertension, angina, heart attack, heart murmur, abnormal heart rhythm, stroke, and high cholesterol. Age that participants left full-time education was categorised as: age 14 or under, 15-16 years, 17-18 years, and age 19 or older. Social class was categorised using the National Statistics Socioeconomic Classification 3 categories;[22] managerial and professional, intermediate, and routine and manual.

Analysis

Independent t-tests were used to compare those with and without diabetes at wave 2 and those who did and did not develop diabetes at follow-up on normally-distributed continuous variables. Mann-Whitney U tests were used for non-normal continuous variables, and Chi-squared tests were used for categorical variables. Spearman rank-order correlations were calculated between all predictor variables and co-variables.

Binary logistic regression was used to test the cross-sectional association of health literacy and cognitive ability with wave 2 diabetes status. Cox regression was used to investigate whether health literacy and cognitive ability test scores at wave 2 predicted risk of developing diabetes between waves 2 and 7. In the Cox regression analysis, time-to-event was taken as the difference, in days, between date of wave 2 and date of diabetes diagnosis for those who self-reported diabetes. For other participants, time-to-event was the difference between date of

For peer review only - http://bmjopen.bmj.com/site/about/guidelines.xhtml

wave 2 interview and the date of last interview. Month and year, but not day, were recorded for date of interview and date of diabetes diagnosis. To create a date variable (yyyy.mm.dd), the day was set to the middle of the month.

For the logistic regressions and Cox regressions, 7 models were run. Age and sex were entered into all models. Health literacy and cognitive ability were entered individually in models 1 and 2, respectively. Both health literacy and cognitive ability were added in Model 3 to determine whether the size of the health literacy-diabetes and cognitive ability-diabetes associations changed when simultaneously entering both these variables. Health literacy and cognitive ability were also entered together in models 4-7. To assess whether BMI and health behaviours accounted for these associations, BMI, smoking status, alcohol consumption, and physical activity were added in Model 4. Diabetes is a risk factor for cardiovascular disease.[23] Associations between poorer cognitive ability and cardiovascular disease are also well established.[24, 25] It is possible that any association between health literacy and cognitive ability with diabetes may be because of these associations with cardiovascular disease. To determine whether any association between health literacy and cognitive ability with diabetes was attenuated when adjusting for cardiovascular disease, number of cardiovascular comorbidities was added in Model 5. Age of leaving full-time education and occupational social class were added in Model 6 to determine whether the association between health literacy, cognitive ability and diabetes was attenuated when accounting for these commonly-used indicators of socioeconomic status. A fully-adjusted model (Model 7) adjusted for health literacy, cognitive ability, and all covariates.

This study was interested in the associations of health literacy and cognitive ability with self-reported diabetes and the independence of these associations with respect to other health and socioeconomic-related variables. In the main text we report the ORs (95% CIs) and the HRs

For peer review only - http://bmjopen.bmj.com/site/about/guidelines.xhtml

(95% CIs) for health literacy and cognitive ability only. The estimates for all covariates entered into the models are reported in the Supplementary materials.

RESULTS

Of the 8,726 ELSA participants who completed wave 2, 3 participants were removed who answered "don't know" to whether a doctor had diagnosed them with diabetes. A further 54 participants were removed because they selected that they had "diabetes or high blood sugar" from a Showcard listing cardiovascular conditions, but, when asked whether a doctor had ever told them they had diabetes, they answered "no". The analytic sample consisted of 8,669 participants. Participant characteristics are reported in Table 1.

Baseline diabetes status

At baseline, 708 (8.2%) participants self-reported a diagnosis of diabetes. Compared to those without diabetes, those with diabetes were more likely to have limited health literacy (42.2% versus 32.3%; $p<0.001$) and have lower cognitive ability (diabetes mean=-0.36, SD=0.97; no diabetes mean=0.03, SD=1.00; Cohen's d=0.40; $p<0.001$). Compared to participants without diabetes, participants with diabetes were older, more likely to be male, leave full-time education at a younger age, be from a less professional social class, have a higher BMI, consume less alcohol, be inactive, and self-report more cardiovascular comorbidities. Rank-order correlations between predictor variables and co-variables are reported in Table 2. Adequate health literacy was moderately correlated with higher cognitive ability (*rho*=0.31, $p<0.001$).

ORs and 95% CIs for the associations between health literacy and cognitive ability with self-reported diabetes at wave 2 are reported in Table 3 (and Supplementary Table S1). A Box-Tidwell test found the assumption of linearity of the logit was violated. Therefore an age-squared term was included in all models, and a squared term for number of cardiovascular

For peer review only - http://bmjopen.bmj.com/site/about/guidelines.xhtml

comorbidities was included in Model 5. Participants with adequate health literacy were 29% less likely to self-report diabetes (Model 1 OR=0.71; 95% CI=0.61 to 0.84). A 1 SD higher cognitive ability was associated with 27% lower odds of self-reported diabetes (Model 2 OR=0.73; 95% CI=0.67 to 0.80). The association between health literacy and diabetes was attenuated by 38% (OR=0.82; 95% CI=0.69 to 0.98) and the association between cognitive ability and diabetes was attenuated by 19% (OR=0.78; 95% CI= 0.70 to 0.86) when entering both health literacy and cognitive ability in Model 3. Both remained significantly associated with diabetes.

BMI and health behaviours were added in Model 4. The associations between health literacy and cognitive ability with diabetes were attenuated and no longer significant. The cognitive ability-diabetes association was not attenuated after adjusting for cardiovascular comorbidities (Model 5) or when adjusting for education and social class (Model 6). Cognitive ability remained significantly associated with diabetes in these models. The association between health literacy and diabetes was slightly attenuated and no longer significant when adjusting for cardiovascular comorbidities (Model 5) and education and social class (Model 6). In the fully adjusted model (Model 7), the size of the associations between health literacy and cognitive ability with diabetes were reduced further and were non-significant.

In the fully-adjusted model (Model 7; Supplementary Table S1) older age, male sex, having a higher BMI, and reporting more cardiovascular comorbidities were associated with higher odds of having diabetes. The association between number of cardiovascular comorbidities and diabetes became less strong as the number of comorbidities increased. Those who reported drinking alcohol at least once per month, rarely, or who never drank alcohol in the last 12 months were more likely to self-report diabetes when compared to those who reported drinking daily/almost daily. Compared to those who reported being physically inactive, those who took

For peer review only - http://bmjopen.bmj.com/site/about/guidelines.xhtml

part in moderate or vigorous physical activity at least once per week were less likely to self-report diabetes.

Risk of incident diabetes

Of the 7,961 participants who did not self-report diabetes at wave 2, 6,961 participants had at least one wave of follow-up between waves 3 and 7. They form the analytic sample for the association between health literacy, cognitive ability and risk of incident diabetes. A total of 506 (7.3%) participants reported a new diagnosis of diabetes between wave 3 and wave 7, whereas 6,455 (92.7%) participants did not. Median time to follow-up was 9.5 years. Mean time to censor was 4.7 years (SD=3.1) for those with diabetes and 7.8 years (SD=2.9) for those not diagnosed with diabetes. Participant characteristics are reported in Table 1. Compared to participants who did not have incident diabetes, those who did were more likely to have limited health literacy (38.8% versus 30.3%, $p<0.001$) and had lower cognitive ability (diabetes mean=-0.04, SD=0.89; no diabetes mean=0.10, SD=0.98, Cohen's $d=0.15$, $p<0.001$) at wave 2. Compared to those who did not develop diabetes, participants who did were older, more likely to be male, have left full-time education at a younger age, be from a less professional social class, smoke, consume less alcohol, be inactive, and to report more cardiovascular comorbidities at wave 2.

The HRs and 95% CIs for the association between health literacy, cognitive ability and risk of diabetes are reported in Table 4 (and Supplementary Table S2). Adequate health literacy at wave 2 was associated with a 36% lower risk of diabetes (Model 1 HR=0.64; 95% CI=0.53 to 0.77). A 1 SD higher cognitive ability at wave 2 was associated with a 23% lower risk of diabetes (Model 2 HR=0.77; 95% CI=0.69 to 0.85). The association between health literacy and risk of diabetes was attenuated by 22% after adjustment for cognitive ability (Model 3 HR=0.72; 95% CI=0.59 to 0.87), and the association between cognitive ability and risk of

For peer review only - http://bmjopen.bmj.com/site/about/guidelines.xhtml

diabetes was attenuated by 9% after adjusting for health literacy (HR=0.79; 95% CI=0.71 to 0.88). Both health literacy and cognitive ability remained significant predictors of diabetes risk.

BMI and health behaviours were added in Model 4. The associations of health literacy and cognitive ability with diabetes risk were further attenuated but remained statistically significant. When adjusting for number of cardiovascular comorbidities, the association between health literacy and cognitive ability with risk of diabetes remained almost unchanged (Model 5) and both remained significantly associated with diabetes risk. Education and social class was added in Model 6. The size of the association between health literacy and cognitive ability with risk of diabetes were slightly reduced but remained statistically significant. In the fully adjusted model (Model 7) the associations between health literacy and cognitive ability and risk of diabetes were further reduced and no longer significant.

In the fully-adjusted model (Model 7; Supplementary Table S2) male participants, those with a higher BMI, current smokers, and those who reported consuming alcohol rarely (compared to daily/almost daily) at wave 2 had increased risk of diabetes. Participants who reported leaving education at age 19 years or older had a lower risk of diabetes compared to those who left at age 14 years or younger.

Sensitivity analysis

There were some missing data. For the cross-sectional analyses, 70% of participants had complete data. For the longitudinal analyses, 75% of participants had complete data. All models were re-run using only participants with complete data on all variables entered into the models. These results are reported in Supplementary Tables S3 and S4. The pattern of associations were generally similar; however, the sizes of the associations tended to be slightly weaker compared to the full sample. For the cross-sectional analysis, health literacy was no longer significantly associated with diabetes status in Model 3 when adjusting for health

For peer review only

literacy and cognitive ability (Supplementary Table S3). For the longitudinal analysis, when adjusting for BMI and health behaviours (Model 4; Supplementary Table S4), health literacy was no longer associated with risk of diabetes.

DISCUSSION

Using a sample of middle-aged and older adults living in England, the present study found that adequate health literacy and better cognitive ability were associated with lower odds of reporting diabetes. These associations were attenuated when health literacy and cognitive ability were entered in the same model, though both independently contributed to diabetes. Adequate health literacy and better cognitive ability, measured at wave 2, were associated with reduced risk of developing diabetes during a median of 9.5 years follow-up. Health literacy and cognitive ability predicted risk of diabetes when examined individually and when examined simultaneously. Health literacy and cognitive ability were no longer associated with cross-sectional diabetes status or with risk of diabetes when adjusting for health behaviours and BMI. Cross-sectional associations between cognitive ability and diabetes status at wave 2, and associations the between health literacy and cognitive ability with risk of developing diabetes during follow-up persisted after adjusting for cardiovascular comorbidities and indicators of socioeconomic status.

Previous cross-sectional studies have found that individuals with lower health literacy are more likely to report having diabetes[7, 8] and longitudinal studies have found that that lower cognitive ability earlier in life is associated with an increased risk of diabetes.[3, 4] The present study is the first longitudinal study to examine whether health literacy is associated with risk of developing diabetes, and the first to examine whether cognitive ability and health literacy have independent associations with diabetes. The results reported here suggest that cognitive

For peer review only - http://bmjopen.bmj.com/site/about/guidelines.xhtml

capabilities and health-related skills, though related, contribute independently to risk of diabetes.

Some have suggested that health literacy variance is mostly overlapping with cognitive ability.[17, 26] If this were true, one would expect the association between health literacy and diabetes to be fully attenuated when adjusting for cognitive ability. This is not what was found here. The association between health literacy and diabetes was only moderately attenuated (by 38% for baseline diabetes status and by 22% for diabetes risk) when adjusting for cognitive ability; moreover, both remained significant predictors of diabetes. The results suggest that only some of the association of health literacy and diabetes was accounted for by cognitive ability. However, the cognitive ability measure used here included four brief cognitive ability tests that assessed memory, executive function and processing speed, and did not include other important domains of cognitive function, such as reasoning, that are known to load highly on general cognitive ability.[27] Some of the unique contribution of health literacy might be residual cognitive capability that was not picked up by the relatively brief measures of cognitive ability used here.[28]

This study was also interested in examining whether health literacy and cognitive ability were associated with diabetes risk independent of other health-related and socioeconomic risk factors for diabetes. The largest attenuation was seen when entering health behaviours and BMI into the models. BMI and health behaviours fully attenuated the relationship between health literacy, cognitive ability and reporting diabetes at baseline, and partly attenuated the relationship between health literacy, cognitive ability and risk of developing diabetes during follow-up. Better cognitive ability has been associated with health promoting behaviours such as following a healthy diet and taking part in regular exercise.[2, 29-31] Whereas some studies have found associations between better health literacy and taking part in health promoting behaviours,[32] others have not.[33] Individuals with higher health literacy and cognitive

For peer review only - http://bmjopen.bmj.com/site/about/guidelines.xhtml

ability might be better equipped with the health-related skills and knowledge, and the general cognitive capabilities (e.g., to plan, reason and learn) needed to take better care of themselves[5, 34] and to follow health advise such as eating well and exercising, which, in turn, could reduce the risk of developing diabetes.[1]

Education also partly attenuated the association between health literacy and cognitive ability with risk of diabetes. The association between better health literacy and cognitive ability with higher levels of education are well established.[6, 35] Education may lead to better cognitive ability and health literacy, which in turn may lead to better health-related skills and lower rates of diabetes.[17] Higher early life cognitive ability has been found to predict later educational attainment.[35] An alternative, but not mutually exclusive, explanation could be that higher cognitive ability may equip an individual with the skills needed to obtain higher educational qualifications. Higher educational attainment, in turn, may lead to better health (and lower risk of diabetes) by, for example, increasing health-related knowledge and decision-making skills.[17]

This study has a number of strengths and limitations. A key strength is that it examined the association of health literacy, cognitive ability and risk of diabetes longitudinally. Another strength is the relatively large sample size. One limitation is that only a subsample of participants had complete data. Those with missing data may be those with the lowest health literacy and cognitive ability scores. ELSA may also suffer from selective attrition such that those with increased risk of diabetes may be less likely to return for follow-up. The results reported here may not generalise to those with the lowest health literacy and/or cognitive ability, and those with the highest risk of diabetes. Diabetes status was self-reported; however, there is a relatively high rate of agreement between self-reported diabetes and fasting blood glucose in ELSA.[36] In a sub-sample of ELSA participants with data on both self-reported diabetes status and fasting blood glucose levels, only 1.7% had undiagnosed diabetes, which is

For peer review only - http://bmjopen.bmj.com/site/about/guidelines.xhtml

much lower than has been found in other cohort studies.[36] The health literacy test used in the current study was a brief, four-item test which had limited variance (67% of participants scored the highest score). Although brief, this test was sensitive enough to predict diabetes risk in the current study, and it has previously been found to predict mortality.[16]

This study found that adequate health literacy and higher cognitive ability were independently associated with lower odds of self-reported diabetes and with reduced risk of developing diabetes during a median of 9.5 years follow-up. Individuals with poor health literacy and/or cognitive ability might lack the health-related skills and knowledge and the cognitive abilities required to look after their health throughout life, which in turn, may increase the risk of diabetes. Future studies should investigate whether interventions designed to improve the knowledge and skills required to better self-manage health reduce the risk of developing diabetes in individuals with low health literacy and cognitive ability.

For peer review only

For peer review only - http://bmjopen.bmj.com/site/about/guidelines.xhtml

Funding This work was supported by the University of Edinburgh Centre for Cognitive Ageing and Cognitive Epidemiology, part of the cross council Lifelong Health and Wellbeing Initiative, funded by the Biotechnology and Biological Sciences Research Council (BBSRC), and Medical Research Council (MRC) (grant number MR/K026992/1).

Conflict of Interest No potential conflicts of interest were reported.

Author Contributions Chloe Fawns-Ritchie contributed to the conception and design of the project, analysed the data, interpreted the data, drafted the initial manuscript, and critically revised the manuscript. Jackie Price contributed to the conception and design of the project, interpreted the data, and critically revised the manuscript. Ian J. Deary contributed to the conception and design of the project, interpreted the data, and critically revised the manuscript.

Acknowledgements We thank our late colleague, John Starr, for his contribution to the conception and design of this study. The authors thank the participants of the ELSA study. We are grateful to the UK Data service for supplying the data.

Data accessibility Anonymised data from the English Longitudinal Study of Ageing is available from the UK Data Service (https://https://www.ukdataservice.ac.uk/).

For peer review only

For peer review only - http://bmjopen.bmj.com/site/about/guidelines.xhtml

REFERENCES

1 Hussain A, Claussen B, Ramachandran A*, et al.* Prevention of type 2 diabetes: a review. *Diabetes Res Clin Pract* 2007;76:317-26.

2 Batty GD, Deary IJ, Macintyre S. Childhood IQ in relation to risk factors for premature mortality in middle-aged persons: the Aberdeen Children of the 1950s study. *J Epidemiol Community Health* 2007;61:241-7.

3 Mõttus R, Luciano M, Starr JM*, et al.* Diabetes and life-long cognitive ability. *J Psychosom Res* 2013;75:275-8.

4 Twig G, Gluzman I, Tirosh A*, et al.* Cognitive function and the risk for diabetes among young men. *Diabetes care* 2014;37:2982-8.

5 Gottfredson LS. Intelligence: is it the epidemiologists' elusive "fundamental cause" of social class inequalities in health? *J Pers Soc Psychol* 2004;86:174-99.

6 Nielsen-Bohlman L, Panzer AM, Kindig DA. *Health Literacy: A Prescription to End Confusion*. Washinton, DC: The National Academies Press 2004.

7 Adams RJ, Appleton SL, Hill CL*, et al.* Risks associated with low functional health literacy in an Australian population. *Med J Aust* 2009;191:530-4.

8 Wolf MS, Gazmararian JA, Baker DW. Health literacy and functional health status among older adults. *Arch Intern Med* 2005;165:1946-52.

9 Caruso R, Magon A, Baroni I*, et al.* Health literacy in type 2 diabetes patients: a systematic review of systematic reviews. *Acta Diabetol* 2018;55:1-12.

10 Marciano L, Camerini AL, Schulz PJ. The Role of Health Literacy in Diabetes Knowledge, Self-Care, and Glycemic Control: a Meta-analysis. *J Gen Intern Med* 2019;34:1007-17.

11 Al Sayah F, Majumdar SR, Williams B*, et al.* Health literacy and health outcomes in diabetes: a systematic review. *J Gen Intern Med* 2013;28:444-52.

For peer review only - http://bmjopen.bmj.com/site/about/guidelines.xhtml

12 Bennett JS, Boyle PA, James BD*, et al.* Correlates of health and financial literacy in older adults without dementia. *BMC Geriatrics* 2012;12:30.

13 Murray C, Johnson W, Wolf MS*, et al.* The Association between Cognitive Ability across the Lifespan and Health Literacy in Old Age: The Lothian Birth Cohort 1936. *Intelligence* 2011;39:178-87.

14 Lamar M, Wilson RS, Yu L*, et al.* Associations of literacy with diabetes indicators in older adults. *J Epidemiol Community Health* 2019;73:250-5.

15 Baker DW, Wolf MS, Feinglass J*, et al.* Health literacy, cognitive abilities, and mortality among elderly persons. *J Gen Intern Med* 2008;23:723-6.

16 Bostock S, Steptoe A. Association between low functional health literacy and mortality in older adults: longitudinal cohort study. *BMJ* 2012;344:e1602.

17 Mõttus R, Johnson W, Murray C*, et al.* Towards understanding the links between health literacy and physical health. *Health Psychol* 2014;33:164-73.

18 Fawns-Ritchie C, Starr JM, Deary IJ. Role of cognitive ability in the association between functional health literacy and mortality in the Lothian Birth Cohort 1936: a prospective cohort study. *BMJ Open* 2018;8:e022502.

19 Steptoe A, Breeze E, Banks J*, et al.* Cohort profile: the English Longitudinal Study of Ageing. *Int J Epidemiol* 2013;42:1640-8.

20 Kobayashi LC, Wardle J, von Wagner C. Limited health literacy is a barrier to colorectal cancer screening in England: evidence from the English Longitudinal Study of Ageing. *Prev Med* 2014;61:100-5.

21 Gale CR, Deary IJ, Wardle J*, et al.* Cognitive ability and personality as predictors of participation in a national colorectal cancer screening programme: the English Longitudinal Study of Ageing. *J Epidemiol Community Health* 2015;69:530-5.

For peer review only - http://bmjopen.bmj.com/site/about/guidelines.xhtml

22 Rose D, Pevalin DJ, O'Reilly K. *The National Statistics Socio-economic Classification: origins, development and use*: Palgrave Macmillan Basingstoke 2005.

23 Sarwar N, Gao P, Seshasai SR*, et al.* Diabetes mellitus, fasting blood glucose concentration, and risk of vascular disease: a collaborative meta-analysis of 102 prospective studies. *Lancet* 2010;375:2215-22.

24 Rostamian S, Mahinrad S, Stijnen T*, et al.* Cognitive impairment and risk of stroke: a systematic review and meta-analysis of prospective cohort studies. *Stroke* 2014;45:1342-8.

25 Hart CL, Taylor MD, Smith GD*, et al.* Childhood IQ and cardiovascular disease in adulthood: prospective observational study linking the Scottish Mental Survey 1932 and the Midspan studies. *Social Science & Medicine* 2004;59:2131-8.

26 Reeve CL, Basalik D. Is health literacy an example of construct proliferation? A conceptual and empirical evaluation of its redundancy with general cognitive ability. *Intelligence* 2014;44:93-102.

27 Salthouse TA. Localizing age-related individual differences in a hierarchical structure. *Intelligence* 2004;32:541-61.

28 Fawns-Ritchie C, Starr JM, Deary IJ. Health literacy, cognitive ability and smoking: a cross-sectional analysis of the English Longitudinal Study of Ageing. *BMJ Open* 2018;8:e023929.

29 Batty GD, Deary IJ, Schoon I*, et al.* Childhood mental ability in relation to food intake and physical activity in adulthood: the 1970 British Cohort Study. *Pediatrics* 2007;119:e38-45.

30 Wraw C, Der G, Gale CR*, et al.* Intelligence in youth and health behaviours in middle age. *Intelligence* 2018;69:71-86.

31 Batty GD, Deary IJ, Schoon I*, et al.* Mental ability across childhood in relation to risk factors for premature mortality in adult life: the 1970 British Cohort Study. *J Epidemiol Community Health* 2007;61:997-1003.

For peer review only - http://bmjopen.bmj.com/site/about/guidelines.xhtml

32 von Wagner C, Knight K, Steptoe A*, et al.* Functional health literacy and health-promoting behaviour in a national sample of British adults. *J Epidemiol Community Health* 2007;61:1086-90.

33 Wolf MS, Gazmararian JA, Baker DW. Health literacy and health risk behaviors among older adults. *Am J Prev Med* 2007;32:19-24.

34 Deary IJ, Weiss A, Batty GD. Intelligence and Personality as Predictors of Illness and Death: How Researchers in Differential Psychology and Chronic Disease Epidemiology Are Collaborating to Understand and Address Health Inequalities. *Psychol Sci Public Interest* 2010;11:53-79.

35 Deary IJ, Strand S, Smith P*, et al.* Intelligence and educational achievement. *Intelligence* 2007;35:13-21.

36 Pierce MB, Zaninotto P, Steel N*, et al.* Undiagnosed diabetes-data from the English longitudinal study of ageing. *Diabetic Med* 2009;26:679-85.

For peer review only

For peer review only - http://bmjopen.bmj.com/site/about/guidelines.xhtml

Table 1 Participant characteristics by diabetes status

	Baseline diabetes status reported at wave 2				Incident diabetes reported at follow-up*			
	n	No diabetes (n = 7961)	Diabetes (n = 708)	p	n	No diabetes (n = 6455)	Diabetes (n = 506)	p
Age, mean (SD)	8669	66.46 (9.70)	69.38 (9.16)	<0.001	6961	66.02 (9.53)	65.51 (8.59)	<0.001
Sex, n (%)	8669			<0.001	6961			<0.001
Male		3522 (44.2%)	379 (53.5%)			2791 (43.2%)	262 (51.8%)	
Female		4439 (55.8%)	329 (46.5%)			3664 (56.8%)	244 (48.2%)	
Age left full-time education, n (%)	8468			<0.001	6809			<0.001
≤14 years		1641 (21.1%)	210 (30.6%)			1222 (19.3%)	107 (21.8%)	
15-16 years		4085 (52.5%)	349 (50.8%)			3283 (52.0%)	302 (61.6%)	
17-18 years		1009 (13.0%)	55 (8.0%)			870 (13.8%)	45 (9.2%)	
≥19 years		1046 (13.4%)	73 (10.6%)			944 (14.9%)	36 (7.3%)	
Social class, n (%)	8508			<0.001	6846			<0.001
Managerial and professional		2444 (31.2%)	194 (28.4%)			2067 (32.6%)	133 (26.7%)	
Intermediate		1979 (25.3%)	131 (19.2%)			1662 (26.2%)	104 (20.9%)	
Routine and manual		3403 (43.5%)	357 (52.3%)			2619 (41.3%)	261 (52.4%)	
Health literacy, n (%)	8293			<0.001	6736			<0.001
Adequate		5172 (67.7%)	376 (57.8%)			4351 (69.7%)	300 (61.2%)	
Limited		2471 (32.3%)	274 (42.2%)			1895 (30.3%)	190 (38.8%)	
Cognitive ability, mean (SD)	8335	0.03 (1.00)	-0.36 (0.97)	<0.001	6746	0.10 (0.98)	-0.04 (0.89)	<0.001
BMI, mean (SD)	7179	27.71 (4.79)	30.45 (5.37)	<0.001	5997	27.46 (4.64)	31.21 (5.28)	<0.001
Current smoker, n (%)	8622			0.377	6929			<0.001
Yes		1216 (15.4%)	99 (14.1%)			934 (14.5%)	105 (20.8%)	
No		6704 (84.6%)	603 (85.9%)			5490 (85.5%)	400 (79.2%)	
Alcohol, n (%)	7577			<0.001	6239			<0.001
Never		723 (10.3%)	112 (19.3%)			565 (9.7%)	49 (11.2%)	
Rarely		1076 (15.4%)	124 (21.3%)			863 (14.9%)	90 (20.6%)	

For peer review only - http://bmjopen.bmj.com/site/about/guidelines.xhtml

At least once a month		827 (11.8%)	85 (14.6%)			669 (11.5%)	70 (16.1%)	
At least once a week		2662 (38.1%)	171 (29.4%)			2255 (38.9%)	149 (34.2%)	
Daily/almost daily		1708 (24.4%)	89 (15.3%)			1451 (25.0%)	78 (17.9%)	
Physical activity, n (%)	8665			< 0.001	6958			<0.001
Vigorous activity		2236 (28.1%)	108 (15.2%)			1938 (30.0%)	116 (22.9%)	
Moderate activity		3888 (48.9%)	305 (43.1%)			3194 (49.5%)	233 (46.0%)	
Inactive		1833 (23.0%)	295 (41.7%)			1320 (20.5%)	157 (31.0%)	
Number of cardiovascular comorbidities, mean (SD)	8669	0.67 (0.91)	1.28 (1.13)	<0.001	6961	0.64 (0.88)	0.89 (1.04)	<0.001

BMI, body mass index.

*Incident diabetes reported at follow-up comparisons are based on a subsample of participants who did not self-report a diagnosis of diabetes at wave 2 and with at least one wave of follow-up.

For peer review only

For peer review only - http://bmjopen.bmj.com/site/about/guidelines.xhtml

Table 2 Spearman rank-order correlations between covariates (n=6,463 to 8,660)

	Age	Sex	Education	Social class	Health literacy	Cognitive ability	BMI	Smoking	Alcohol	Physical activity	CV comorbid
Age											
Sex	-0.03**										
Education	-0.41***	0.02									
Social class	0.08***	-0.09***	-0.41***								
Health literacy	-0.16***	0.01	0.23***	-0.18***							
Cognitive ability	-0.47***	-0.09***	0.39***	-0.27***	0.31***						
BMI	-0.07***	0.02	-0.06***	0.08***	-0.04**	-0.01					
Smoking	-0.13***	0.01	-0.05***	0.12***	-0.04***	-0.02	-0.09***				
Alcohol	-0.11***	0.21***	0.22***	-0.20***	0.09***	0.16***	-0.11***	-0.04***			
Physical activity	-0.26***	0.10***	0.23***	-0.15***	0.14***	0.26***	-0.11***	-0.09***	0.18***		
CV comorbid	0.18***	0.00	-0.11***	0.05***	-0.06***	-0.11***	0.14***	-0.03*	-0.08***	-0.14***	

*p<.05, **p<.01, ***p<.001

BMI, body mass index; CV comorbid, number of cardiovascular comorbidities.

Sex is coded 0 for female, 1 for male; Education is age of leaving full-time education and is coded 1 for age 14 years or less, 2 for age 15-16 years, 3 for age 17-18 years, and 4 for 19 years or older; Social class is coded 1 for managerial and professional, 2 for intermediate, and 3 for routine and manual; Health literacy is coded 0 for limited and 1 for adequate; Smoking is coded 0 for current non-smoker and 1 for current smoker; Alcohol is the frequency of alcohol consumed in the last 12 months and is coded 0 for never, 1 for rarely, 2 for at least once a month, 3 for at least once a week, 4 for daily/almost daily; Physical activity is coded 0 for inactive, 1 for moderate activity at least once per week, 2 for vigorous activity at least once per week; CV comorbid is the number of cardiovascular comorbidities self-reported from hypertension, angina, heart attack, heart failure, heart murmur, abnormal heart rhythm, stroke, and high cholesterol.

For peer review only

For peer review only - http://bmjopen.bmj.com/site/about/guidelines.xhtml

Table 3 Odds ratios (95% CI) from logistic regression models of the association between health literacy and cognitive ability with self-reported diabetes at wave 2

	Model 1: Health literacy	Model 2: Cognitive ability	Model 3: Health literacy and cognitive ability	Model 4: +BMI and health behaviours	Model 5: +CV comorbidities	Model 6: +Education and social class	Model 7: Fully-adjusted
Adequate health literacy	0.71*** (0.61, 0.84)	-	0.82* (0.69, 0.98)	0.97 (0.78, 1.21)	0.85 (0.72, 1.02)	0.84 (0.70, 1.01)	0.98 (0.78, 1.23)
Cognitive ability	-	0.73*** (0.67, 0.80)	0.78*** (0.70, 0.86)	0.90 (0.80, 1.02)	0.78*** (0.71, 0.87)	0.78*** (0.71, 0.87)	0.87 (0.76, 1.00)

*p<.05, **p<.01, ***p<.001

BMI, body mass index; CV, cardiovascular.

All models adjusted for age, age-squared, and sex. Models 1 n=8,293, Model 2 n=8,335, Model 3 n=8,185. Model 4 (n=6,302) adjusted for body mass index, frequency of alcohol consumption in the past 12 months, and physical activity. Model 5 (n=8,185) adjusted for number of cardiovascular comorbidities reported, and a squared term for number of cardiovascular comorbidities reported. Model 6 (n=7,861) adjusted for age left full-time education, and occupational social class. Model 7 (n=6,086) adjusted for all covariates.

For peer review only - http://bmjopen.bmj.com/site/about/guidelines.xhtml

Table 4 Hazard ratios (95% CI) from Cox regression models of the association between health literacy and cognitive ability with risk of incident diabetes

	Model 1: Health literacy	Model 2: Cognitive ability	Model 3: Health literacy and cognitive ability	Model 4: +BMI and health behaviours	Model 5: +CV comorbidities	Model 6: +Education and social class	Model 7: Fully-adjusted
Adequate health literacy	0.64*** (0.53, 0.77)	-	0.72*** (0.59, 0.87)	0.79* (0.64, 0.99)	0.73** (0.60, 0.88)	0.79* (0.65, 0.97)	0.85 (0.68, 1.06)
Cognitive ability	-	0.77*** (0.69, 0.85)	0.79*** (0.71, 0.88)	0.85* (0.74, 0.96)	0.80*** (0.71, 0.89)	0.84** (0.75, 0.95)	0.88 0.77, 1.01)

*p<.05, **p<.01, ***p<.001

BMI, body mass index, CV, cardiovascular.

All models adjusted for age and sex. Models 1 (n=6,736) had 490 diabetes events, Model 2 (n=6,746) had 491 diabetes events, Model 3 (n=6,654) had 484 diabetes events. Model 4 (n=5,357; 377 diabetes events) adjusted for body mass index, frequency of alcohol consumption in the past 12 months, and physical activity. Model 5 (n=6,654; 484 diabetes events) adjusted for number of cardiovascular comorbidities reported. Model 6 (n=6,409; 492 diabetes events) adjusted for age left full-time education, and occupational social class. Model 7 (n=5,186, 360 diabetes events) adjusted for all covariates.

For peer review only - http://bmjopen.bmj.com/site/about/guidelines.xhtml

Supplementary material for:

The association of health literacy and cognitive ability with self-reported diabetes in the English Longitudinal Study of Ageing

For peer review only

For peer review only - http://bmjopen.bmj.com/site/about/guidelines.xhtml

Supplementary Table S1 Odds ratios (95% CI) from logistic regression models of the association between health literacy and cognitive ability with self-reported diabetes at wave 2

	Model 1: Health literacy n=8,293	Model 2: Cognitive ability n=8,335	Model 3: Health literacy and cognitive ability n=8,185	Model 4: +BMI and health behaviours n=6,302	Model 5: +CV comorbidities n=8,185	Model 6: +Education and social class n=7,861	Model 7: Fully-adjusted n=6,086
Health literacy							
Limited	Reference	-	Reference	Reference	Reference	Reference	Reference
Adequate	0.71***		0.82*	0.97	0.85	0.84	0.98
	(0.61, 0.84)		(0.69, 0.98)	(0.78, 1.21)	(0.72, 1.02)	(0.70, 1.01)	(0.78, 1.23)
Cognitive ability	-	0.73***	0.78***	0.90	0.78***	0.78***	0.87
		(0.67, 0.80)	(0.70, 0.86)	(0.80, 1.02)	(0.71, 0.87)	(0.71, 0.87)	(0.76, 1.00)
Age	1.04***	1.03***	1.03***	1.04***	1.02***	1.03***	1.03***
	(1.03, 1.05)	(1.02, 1.04)	(1.02, 1.04)	(1.03, 1.06)	(1.01, 1.03)	(1.02, 1.04)	(1.02, 1.05)
Age^2	0.998***	0.998***	0.998***	0.998**	0.998***	0.998***	0.999
	(0.997, 0.999)	(0.997, 0.998)	(0.997, 0.999)	(0.997, 0.999)	(0.997, 0.999)	(0.997, 0.999)	(0.998, 1.000)
Sex							
Female	Reference	Reference	Reference	Reference	Reference	Reference	Reference
Male	1.50***	1.41***	1.43	2.16***	1.45***	1.44***	2.09***
	(1.28, 1.77)	(1.20, 1.66)	(1.22, 1.69)	(1.75, 2.68)	(1.23, 1.71)	(1.22, 1.71)	(1.67, 2.62)
BMI				1.10***			1.09***
				(1.08, 1.12)			(1.07, 1.11)
Current smoking							
Non-smoker				Reference			Reference
Smoker				0.91			0.93
				(0.66, 1.23)			(0.66, 1.27)
Alcohol consumption							
Daily/almost daily				Reference			Reference
At least once per week				1.21			1.24
				(0.90, 1.65)			(0.91, 1.70)
At least once per month				1.78**			1.77**
				(1.24, 2.56)			(1.21, 2.57)
Rarely				1.95***			1.95***
				(1.38, 2.76)			(1.36, 2.79)
Never				2.40***			2.12***
				(1.67, 3.44)			(1.45, 3.11)
Physical activity							
Inactive				Reference			Reference

For peer review only

For peer review only - http://bmjopen.bmj.com/site/about/guidelines.xhtml

Moderate activity	0.65*** (0.51, 0.83)			0.68** (0.53, 0.87)
Vigorous activity	0.50*** (0.37, 0.68)			0.56*** (0.41, 0.76)
Number of CV comorbidities		2.08*** (1.84, 2.36)		1.98*** (1.70, 2.32)
Number of CV comorbidities^2		0.88*** (0.84, 0.93)		0.88*** (0.82, 0.93)
Age left full-time education				
≤14 years			Reference	Reference
15-16 years			1.06 (0.84, 1.34)	1.16 (0.87, 1.56)
17-18 years			0.81 (0.56, 1.14)	0.98 (0.63, 1.50)
≥19 years			1.06 (0.74, 1.50)	1.32 (0.85, 2.05)
Social class				
Managerial and professional			Reference	Reference
Intermediate			0.79 (0.61, 1.02)	0.79 (0.58, 1.07)
Routine and manual			1.08 (0.87, 1.35)	1.01 (0.77, 1.32)

*p<.05, **p<.01, ***p<.001

Age^2, age squared; BMI, body mass index; CV, cardiovascular; number of CV cormorbidities^2, number of cardiovascular comorbidities squared.

For peer review only

Supplementary Table S2 Hazard ratios (95% CI) from Cox regression models of the association between health literacy and cognitive ability with risk of diabetes

	Model 1: Health literacy n=6,736 Events=490	Model 2: Cognitive ability n=6,746 Events=491	Model 3: Health literacy and cognitive ability n=6,654 Events=484	Model 4: +BMI health behaviours n=5,357 Events=377	Model 5: +CV comorbidities n=6654 Events=484	Model 6: +Education and social class n=6409 Events=462	Model 7: Fully-adjusted n=5,186 Events=360
Health literacy							
Limited	Reference	-	Reference	Reference	Reference	Reference	Reference
Adequate	0.64*** (0.53, 0.77)		0.72*** (0.59, 0.87)	0.79* (0.64, 0.99)	0.73** (0.60, 0.88)	0.79* (0.65, 0.97)	0.85 (0.68, 1.06)
Cognitive ability	-	0.77*** (0.69, 0.85)	0.79*** (0.71, 0.88)	0.85* (0.74, 0.96)	0.80*** (0.71, 0.89)	0.84** (0.75, 0.95)	0.88 (0.77, 1.01)
Age	1.01 (1.00, 1.02)	1.00 (0.99, 1.01)	1.00 (0.98, 1.01)	1.01 (1.00, 1.02)	0.99 (0.98, 1.00)	1.00 (0.98, 1.01)	1.01 (0.99, 1.02)
Sex							
Female	Reference	Reference	Reference	Reference	Reference	Reference	Reference
Male	1.43*** (1.20, 1.71)	1.39*** (1.16, 1.66)	1.38*** (1.15, 1.65)	1.84*** (1.49, 2.29)	1.38*** (1.16, 1.66)	1.39*** (1.15, 1.68)	1.82*** (1.45, 2.28)
BMI				1.12*** (1.10, 1.14)			1.12*** (1.10, 1.13)
Current smoking							
Non-smoker				Reference			Reference
Smoker				1.77*** (1.35, 2.31)			1.69*** (1.28, 2.22)
Alcohol consumption							
Daily/almost daily				Reference			Reference
At least once per week				1.11 (0.83, 1.49)			1.01 (0.75, 1.37)
At least once per month				1.53* (1.07, 2.19)			1.40 (0.97, 2.01)
Rarely				1.78*** (1.27, 2.50)			1.53* (1.08, 2.17)
Never				1.42 (0.95, 2.11)			1.15 (0.76, 1.73)
Physical activity							
Inactive				Reference			Reference
Moderate activity				0.78			0.79

For peer review only - http://bmjopen.bmj.com/site/about/guidelines.xhtml

	(0.61, 1.01)			(0.61, 1.03)
Vigorous activity	0.72* (0.54, 0.98)			0.76 (0.56, 1.04)
Number of CV comorbidities		1.34*** (1.22, 1.46)		1.17** (1.05, 1.30)
Age left full-time education				
≤14 years			Reference	Reference
15-16 years			0.93 (0.71, 1.22)	1.00 (0.74, 1.36)
17-18 years			0.61* (0.41, 0.91)	0.73 (0.47, 1.15)
≥19 years			0.44*** (0.28, 0.68)	0.58* (0.35, 0.96)
Social class				
Managerial and professional			Reference	Reference
Intermediate			0.81 (0.62, 1.07)	0.91 (0.66, 1.24)
Routine and manual			1.17 (0.93, 1.49)	1.17 (0.89, 1.53)

*p<.05, **p<.01, ***p<.001

BMI, body mass index; CV, cardiovascular.

For peer review only - http://bmjopen.bmj.com/site/about/guidelines.xhtml

Supplementary Table S3 Odds ratios (95% CI) for the association between health literacy and cognitive ability with cross-sectional diabetes status at wave 2 in a sub-sample of 6,086 participants with data on all variables of interest.

	Model 1: Health literacy	Model 2: Cognitive ability	Model 3: Health literacy and cognitive ability	Model 4: +BMI and health behaviours	Model 5: +CV comorbidities	Model 6: +Education and social class	Model 7: Fully-adjusted
Health literacy							
Limited	Reference	-	Reference	Reference	Reference	Reference	Reference
Adequate	0.79* (0.64, 0.97)		0.88 (0.71, 1.10)	0.96 (0.77, 1.20)	0.92 (0.74, 1.15)	0.90 (0.72, 1.12)	0.98 (0.78, 1.23)
Cognitive ability	-	0.78*** (0.69, 0.88)	0.79*** (0.70, 0.90)	0.88 (0.77, 1.00)	0.80*** (0.70, 0.91)	0.82** (0.72, 0.93)	0.88 (0.77, 1.00)
Age	1.04*** (1.03, 1.06)	1.03*** (1.02, 1.05)	1.03*** (1.02, 1.05)	1.04*** (1.02, 1.05)	1.02** (1.01, to 1.04)	1.04*** (1.02, 1.05)	1.03*** (1.02, 1.05)
Age^2	0.999* (0.997, 1.000)	0.999** (0.997, 1.000)	0.999** (0.997, 1.000)	0.998** (0.997, 1.000)	0.999 (0.998, 1.000)	0.999* (0.997, 1.000)	0.999 (0.998, 1.000)
Sex							
Female	Reference	Reference	Reference	Reference	Reference	Reference	Reference
Male	1.66*** (1.36, 2.03)	1.58*** (1.29, 1.93)	1.58*** (1.29, 1.94)	2.17*** (1.74, 2.70)	1.63*** (1.33, 2.00)	1.56 (1.27, 1.92)	2.09*** (1.67, 2.62)
BMI				1.10*** (1.08, 1.12)			1.09*** (1.07, 1.11)
Current smoking							
Non-smoker				Reference			Reference
Smoker				0.89 (0.64, 1.22)			0.93 (0.66, 1.27)
Alcohol consumption							
Daily/almost daily				Reference			Reference
At least once per week				1.21 (0.90, 1.66)			1.24 (0.91, 1.70)
At least once per month				1.76** (1.21, 2.54)			1.77** (1.21, 2.57)
Rarely				2.01*** (1.42, 2.87)			1.95*** (1.36, 2.79)
Never				2.24*** (1.55, 3.26)			2.12*** (1.45, 3.11)
Physical activity							
Inactive				Reference			Reference
Moderate activity				0.65***			0.68**

For peer review only

	(0.51, 0.82)			(0.53, 0.87)
Vigorous activity	0.51***			0.56***
	(0.37, 0.69)			(0.41, 0.76)
Number of CV comorbidities		2.22***		1.98***
		(1.91, 2.59)		(1.70, 2.32)
Number of CV comorbidities^2		0.87***		0.88***
		(0.81, 0.92)		(0.82, 0.93)
Education				
≤14 years			Reference	Reference
15-16 years			1.07	1.17
			(0.81, 1.42)	(0.87, 1.56)
17-18 years			0.78	0.98
			(0.51, 1.18)	(0.64, 1.50)
≥19 years			0.94	1.32
			(0.62, 1.43)	(0.85, 2.05)
Social class				
Managerial and professional			Reference	Reference
Intermediate			0.79	0.79
			(0.59, 1.07)	(0.58, 1.07)
Routine and manual			1.09	1.01
			(0.84, 1.42)	(0.77, 1.32)

*$p<.05$, **$p<.01$, ***$p<.001$

Age^2, age squared; BMI, body mass index; CV, cardiovascular; number of CV cormorbidities^2, number of cardiovascular comorbidities squared.

For peer review only

Supplementary Table S4 Hazard ratios (95% CIs) from Cox regression models of the association between health literacy and cognitive ability with risk of diabetes. Models are run on a sub-sample of 5,186 (360 with incident diabetes) participants with data on all variables of interest.

	Model 1: Health literacy	Model 2: Cognitive ability	Model 3: Health literacy and cognitive ability	Model 4: +BMI and health behaviours	Model 5: +CV comorbidities	Model 6: +Education and social class	Model 7: Fully-adjusted
Health literacy							
Limited	Reference	-	Reference	Reference	Reference	Reference	Reference
Adequate	0.64*** (0.52, 0.80)		0.73** (0.58, 0.91)	0.80 (0.64, 1.01)	0.74** (0.59, 0.93)	0.79* (0.63, 0.98)	0.85 (0.68, 1.06)
Cognitive ability	-	0.72*** (0.63, 0.82)	0.76*** (0.66, 0.86)	0.84** (0.73, 0.96)	0.76*** (0.67, 0.87)	0.83** (0.72, 0.95)	0.88 (0.77, 1.01)
Age	1.01 (0.997, 1.02)	1.00 (0.98, 1.01)	1.00 (0.98, 1.01)	1.01 (0.997, 1.03)	0.99 (0.98, 1.01)	1.00 (0.98, 1.01)	1.01 (0.99, 1.02)
Sex							
Female	Reference	Reference	Reference	Reference	Reference	Reference	Reference
Male	1.47*** (1.20, 1.81)	1.38** (1.12, 1.70)	1.40** (1.13, 1.72)	1.82*** (1.46, 2.27)	1.40** (1.14, 1.73)	1.42** (1.15, 1.76)	1.82*** (1.45, 2.28)
BMI				1.12*** (1.10, 1.14)			1.12*** (1.10, 1.13)
Current smoking							
Non-smoker				Reference			Reference
Smoker				1.79*** (1.36, 2.34)			1.69*** (1.28, 2.22)
Alcohol consumption							
Daily/almost daily				Reference			Reference
At least once per week				1.10 (0.80, 1.46)			1.01 (0.75, 1.36)
At least once per month				1.49* (1.03, 2.14)			1.40 (0.97, 2.01)
Rarely				1.70** (1.20, 2.40)			1.53* (1.08, 2.17)
Never				1.30 (0.86, 1.96)			1.15 (0.76, 1.73)
Physical activity							
Inactive				Reference			Reference
Moderate activity				0.76* (0.59, 0.99)			0.79 (0.61, 1.03)
Vigorous activity				0.71*			0.76

For peer review only - http://bmjopen.bmj.com/site/about/guidelines.xhtml

	(0.52, 0.96)			(0.56, 1.04)
Number of CV comorbidities		1.30***		1.17**
		(1.17, 1.45)		(1.05, 1.30)
Education				
≤14 years			Reference	Reference
15-16 years			0.90	1.00
			(0.67, 1.23)	(0.74, 1.36)
17-18 years			0.56*	0.73
			(0.36, 0.88)	(0.47, 1.15)
≤19 years			0.44**	0.58*
			(0.27, 0.73)	(0.35, 0.96)
Social class				
Managerial and professional			Reference	Reference
Intermediate			0.86	0.91
			(0.63, 1.17)	(0.66, 1.24)
Routine and manual			1.24	1.16
			(0.95, 1.63)	(0.89, 1.53)

*$p<.05$, **$p<.01$, ***$p<.001$

BMI, body mass index; CV, cardiovascular.

For peer review only - http://bmjopen.bmj.com/site/about/guidelines.xhtml

STROBE Statement—checklist of items that should be included in reports of observational studies

	Item No	Recommendation	
**Title and abstract**	1	(*a*) Indicate the study's design with a commonly used term in the title or the abstract	Page 1
		(*b*) Provide in the abstract an informative and balanced summary of what was done and what was found	Page 2
**Introduction**			
Background/rationale	2	Explain the scientific background and rationale for the investigation being reported	Pages 5-6
Objectives	3	State specific objectives, including any prespecified hypotheses	Page 6
**Methods**			
Study design	4	Present key elements of study design early in the paper	Pages 6-8
Setting	5	Describe the setting, locations, and relevant dates, including periods of recruitment, exposure, follow-up, and data collection	Pages 6-7
Participants	6	(*a*) *Cohort study*—Give the eligibility criteria, and the sources and methods of selection of participants. Describe methods of follow-up<br>*Case-control study*—Give the eligibility criteria, and the sources and methods of case ascertainment and control selection. Give the rationale for the choice of cases and controls<br>*Cross-sectional study*—Give the eligibility criteria, and the sources and methods of selection of participants	Pages 6-7
		(*b*) *Cohort study*—For matched studies, give matching criteria and number of exposed and unexposed<br>*Case-control study*—For matched studies, give matching criteria and the number of controls per case	NA
Variables	7	Clearly define all outcomes, exposures, predictors, potential confounders, and effect modifiers. Give diagnostic criteria, if applicable	Pages 7-10
Data sources/ measurement	8*	For each variable of interest, give sources of data and details of methods of assessment (measurement). Describe comparability of assessment methods if there is more than one group	Pages 7-10
Bias	9	Describe any efforts to address potential sources of bias	Pages 15-16
Study size	10	Explain how the study size was arrived at	Pages 7, 12
Quantitative variables	11	Explain how quantitative variables were handled in the analyses. If applicable, describe which groupings were chosen and why	Pages 10-11
Statistical methods	12	(*a*) Describe all statistical methods, including those used to control for confounding	Pages 10-11
		(*b*) Describe any methods used to examine subgroups and interactions	NA
		(*c*) Explain how missing data were addressed	Pages 15-16
		(*d*) *Cohort study*—If applicable, explain how loss to follow-up was addressed<br>*Case-control study*—If applicable, explain how matching of cases and controls was addressed<br>*Cross-sectional study*—If applicable, describe analytical methods taking	Pages 15-16

For peer review only

		account of sampling strategy	
		(*e*) Describe any sensitivity analyses	Pages 15-16
**Results**			
Participants	13*	(a) Report numbers of individuals at each stage of study—eg numbers potentially eligible, examined for eligibility, confirmed eligible, included in the study, completing follow-up, and analysed	Pages 12, 14
		(b) Give reasons for non-participation at each stage	Pages 12, 14
		(c) Consider use of a flow diagram	NA
Descriptive data	14*	(a) Give characteristics of study participants (eg demographic, clinical, social) and information on exposures and potential confounders	Pages 12, 14, Table 1
		(b) Indicate number of participants with missing data for each variable of interest	Table 1
		(c) *Cohort study*—Summarise follow-up time (eg, average and total amount)	Pages 7-8
Outcome data	15*	*Cohort study*—Report numbers of outcome events or summary measures over time	Tables 3 and 4
		Case-control study—Report numbers in each exposure category, or summary measures of exposure	
		Cross-sectional study—Report numbers of outcome events or summary measures	Table 3 and 4
Main results	16	(*a*) Give unadjusted estimates and, if applicable, confounder-adjusted estimates and their precision (eg, 95% confidence interval). Make clear which confounders were adjusted for and why they were included	Tables 1-4
		(*b*) Report category boundaries when continuous variables were categorized	
		(*c*) If relevant, consider translating estimates of relative risk into absolute risk for a meaningful time period	NA
Other analyses	17	Report other analyses done—eg analyses of subgroups and interactions, and sensitivity analyses	Pages 7-10
**Discussion**			
Key results	18	Summarise key results with reference to study objectives	Page 16
Limitations	19	Discuss limitations of the study, taking into account sources of potential bias or imprecision. Discuss both direction and magnitude of any potential bias	Pages 18-19
Interpretation	20	Give a cautious overall interpretation of results considering objectives, limitations, multiplicity of analyses, results from similar studies, and other relevant evidence	Pages 16-19
Generalisability	21	Discuss the generalisability (external validity) of the study results	Pages 18-19
**Other information**			
Funding	22	Give the source of funding and the role of the funders for the present study and, if applicable, for the original study on which the present article is based	Page 20

*Give information separately for cases and controls in case-control studies and, if applicable, for exposed and unexposed groups in cohort and cross-sectional studies.

For peer review only - http://bmjopen.bmj.com/site/about/guidelines.xhtml

Note: An Explanation and Elaboration article discusses each checklist item and gives methodological background and published examples of transparent reporting. The STROBE checklist is best used in conjunction with this article (freely available on the Web sites of PLoS Medicine at http://www.plosmedicine.org/, Annals of Internal Medicine at http://www.annals.org/, and Epidemiology at http://www.epidem.com/). Information on the STROBE Initiative is available at www.strobe-statement.org.

For peer review only

For peer review only - http://bmjopen.bmj.com/site/about/guidelines.xhtml

The association of functional health literacy and cognitive ability with self-reported diabetes in the English Longitudinal Study of Ageing: A prospective cohort study

Journal:	*BMJ Open*
Manuscript ID	bmjopen-2021-058496.R1
Article Type:	Original research
Date Submitted by the Author:	23-Mar-2022

Complete List of Authors:	Fawns-Ritchie, Chloe; The University of Edinburgh, Department of Psychology<br>Price, Jackie; The University of Edinburgh, Usher Institute<br>Deary, Ian; The University of Edinburgh, Department of Psychology

**Primary Subject Heading**:	Epidemiology
Secondary Subject Heading:	Diabetes and endocrinology, Public health
Keywords:	General diabetes < DIABETES & ENDOCRINOLOGY, EPIDEMIOLOGY, Diabetes & endocrinology < INTERNAL MEDICINE, PUBLIC HEALTH

SCHOLARONE™
Manuscripts

I, the Submitting Author has the right to grant and does grant on behalf of all authors of the Work (as defined in the below author licence), an exclusive licence and/or a non-exclusive licence for contributions from authors who are: i) UK Crown employees; ii) where BMJ has agreed a CC-BY licence shall apply, and/or iii) in accordance with the terms applicable for US Federal Government officers or employees acting as part of their official duties; on a worldwide, perpetual, irrevocable, royalty-free basis to BMJ Publishing Group Ltd ("BMJ") its licensees and where the relevant Journal is co-owned by BMJ to the co-owners of the Journal, to publish the Work in this journal and any other BMJ products and to exploit all rights, as set out in our licence.

The Submitting Author accepts and understands that any supply made under these terms is made by BMJ to the Submitting Author unless you are acting as an employee on behalf of your employer or a postgraduate student of an affiliated institution which is paying any applicable article publishing charge ("APC") for Open Access articles. Where the Submitting Author wishes to make the Work available on an Open Access basis (and intends to pay the relevant APC), the terms of reuse of such Open Access shall be governed by a Creative Commons licence – details of these licences and which Creative Commons licence will apply to this Work are set out in our licence referred to above.

Other than as permitted in any relevant BMJ Author's Self Archiving Policies, I confirm this Work has not been accepted for publication elsewhere, is not being considered for publication elsewhere and does not duplicate material already published. I confirm all authors consent to publication of this Work and authorise the granting of this licence.

For peer review only

The association of functional health literacy and cognitive ability with self-reported diabetes in the English Longitudinal Study of Ageing: A prospective cohort study

Chloe Fawns-Ritchie[1,2], Jackie Price[3], and Ian J Deary[1,2]

[1]Department of Psychology, University of Edinburgh, Edinburgh, UK

[2]Centre for Cognitive Ageing and Cognitive Epidemiology, University of Edinburgh, Edinburgh, UK

[3]Usher Institute, University of Edinburgh, Edinburgh, UK

Corresponding author:

Chloe Fawns-Ritchie

Department of Psychology, University of Edinburgh

7 George Square

Edinburgh, EH8 9JZ

Scotland, UK

Telephone: +44 131 650 8317

c.fawns-ritchie@ed.ac.uk

For peer review only

Abstract word count: 289

Manuscript word count: 6,137

For peer review only - http://bmjopen.bmj.com/site/about/guidelines.xhtml

ABSTRACT

Objectives: We investigated whether functional health literacy and cognitive ability were associated with self-reported diabetes.

Design: Prospective cohort study.

Setting: Data was from waves 2 (2004-05) to 7 (2014-15) of the English Longitudinal Study of Ageing (ELSA), a cohort study designed to be representative of adults aged 50 years and older living in England.

Participants: 8,669 ELSA participants (mean age=66.7, SD=9.7) who completed a brief functional health literacy test assessing health-related reading comprehension, and 4 cognitive tests assessing declarative memory, processing speed and executive function at wave 2.

Primary outcome measure: Self-reported doctor diagnosis of diabetes.

Results: Logistic regression was used to examine cross-sectional (wave 2) associations of functional health literacy and cognitive ability with diabetes status. Adequate (compared to limited) functional health literacy (OR=0.72, 95% CI=0.61 to 0.84) and higher cognitive ability (OR per 1 SD=0.73, 95% CI=0.67 to 0.80) were associated with lower odds of self-reporting diabetes at wave 2. Cox regression was used to test the associations of functional health literacy and cognitive ability measured at wave 2 with self-reporting diabetes over a median of 9.5 years follow-up (n=6,961). Adequate functional health literacy (HR=0.64; 95% CI=0.53 to 0.77) and higher cognitive ability (HR=0.77, 95% CI=0.69 to 0.85) at wave 2 were associated with lower risk of self-reporting diabetes during follow-up. When both functional health literacy and cognitive ability were added to the same model, these associations were slightly attenuated. Additionally adjusting for health behaviours and body mass index fully attenuated cross-sectional associations between functional health literacy and cognitive ability with

For peer review only

diabetes status, and partly attenuated associations between functional health literacy and cognitive ability with self-reporting diabetes during follow-up.

Conclusions: Adequate functional health literacy and better cognitive ability were independently associated with lower likelihood of reporting diabetes.

Keywords Health literacy, cognition, diabetes, prospective studies

For peer review only

For peer review only - http://bmjopen.bmj.com/site/about/guidelines.xhtml

Strengths and limitations of this study

- This study used data from the English Longitudinal Study of Ageing, a large prospective cohort study designed to be representative of community-dwelling adults aged over 50 years living in England.
- Participants were followed-up for a median of 9.5 years to determine whether they were diagnosed with diabetes.
- Diabetes status was self-reported.
- Health literacy and cognitive ability assessments were brief.

For peer review only

For peer review only - http://bmjopen.bmj.com/site/about/guidelines.xhtml

INTRODUCTION

Diabetes is a common chronic condition in older adulthood and is associated with substantial morbidity and mortality.[1] Type 2 diabetes, the most common type of diabetes, is at least partly preventable.[1] Understanding the characteristics of those most at risk of developing diabetes is important for appropriately targeting diabetes education and interventions. Risk factors for developing diabetes include older age, deprivation, and obesity.[1]

Lower cognitive ability may be a risk factor for diabetes. Cognitive ability can be conceptualised as a composite term for a range of different but overlapping mental capabilities, including the ability to learn, plan, problem solve and process information.[2] Cognitive ability is closely related to but distinct from educational attainment and correlations between cognitive ability and education range from 0.40 to 0.80.[3] This general mental capability has been found to be associated with many different aspects of health.[2] Studies examining the association between cognitive ability and diabetes have found mixed results. One study[4] found that childhood cognitive ability did not predict diabetes in midlife when individually adjusting for a range of demographic variables including education. Others have found that lower cognitive ability in early life was associated with higher risk of diabetes in adulthood.[5, 6] Whereas the first study[5] did not adjust for educational attainment or measures of socioeconomic status, the latter[6] found that individuals with lower cognitive ability in early adulthood had higher rates of diabetes in midlife, even after adjusting for education and indicators of socioeconomic status. Individuals with higher cognitive ability might have the cognitive skills required to self-manage their health, take better care of themselves throughout life, and thus reduce the risk of developing diabetes.[2, 5]

Health literacy is the “capacity to obtain, process and understand basic health information and services needed to make basic health decisions”[7], and it might also play a role in diabetes.

For peer review only

Health literacy is a multifaceted construct thought to encompass all of the skills required to make decisions about one's health, including the ability to access, appraise and apply health information.[8, 9] One component of health literacy is functional health literacy – the reading, writing and numeracy skills needed to understand basic health information.[10] These skills are thought to be required, for example, to understand and correctly follow the instructions on a packet of prescription medication. In cross-sectional studies, rates of diabetes are higher in those with low functional health literacy, even after adjusting for age, sex, income and education.[11, 12] In one study, participants with inadequate functional health literacy were 48% more likely to report having diabetes when compared to participants with adequate health literacy, adjusting for sociodemographic and health variables.[12] Associations between health literacy and diabetes may differ by sex. Women with low health literacy were found to be more than twice as likely to have diabetes compared to those with high literacy after adjusting for age, race, income, education, body mass index (BMI), and smoking and alcohol status, however, health literacy was not associated with diabetes status in men.[13] Individuals with lower functional health literacy – at least in women – might lack the health-related reading and writing skills required to obtain, understand and follow health advice, such as eating well and exercising, which might reduce the risk of diabetes.[7]

In patients with diabetes, higher functional health literacy has consistently been associated with greater diabetes knowledge.[14-16] A very small association between higher functional health literacy and lower glycated haemoglobin (HbA_{1C}) levels in patients with diabetes has been reported in a meta-analysis of 26 studies (r=-0.048, p=0.027).[15] Whereas studies have investigated the association between functional health literacy and disease management in people with diabetes, little is known about whether functional health literacy is associated with risk of developing diabetes.

For peer review only - http://bmjopen.bmj.com/site/about/guidelines.xhtml

Functional health literacy and cognitive ability test scores are positively correlated.[17-19] Rank-order correlations between general cognitive ability and three functional health literacy tests ranged from 0.37 to 0.50.[18] Researchers have sought to determine the role of cognitive ability in the association between functional health literacy and a range of health outcomes. Most (but not all)[20] studies have found that cognitive ability partly or entirely attenuates the association between functional health literacy and health.[21-24]. One study[19] sought to determine whether health literacy and cognitive ability had independent associations with performance on various health-related tasks, including comprehending written and video-presented health information and using health-related props, such as a pill bottle. Using three different measures of functional health literacy, the association between functional health literacy and performance on the health-related tasks were attenuated by between 70.6% and 77.7% when including cognitive ability in the same model compared to models not including cognitive ability.[19] Any association between functional health literacy and diabetes may be attenuated when also measuring cognitive ability.

The aim of the current study was to better understand the associations of functional health literacy and cognitive ability with diabetes. Using data from the English Longitudinal Study of Ageing (ELSA),[25] the present study investigated whether functional health literacy and cognitive ability were independently associated with diabetes. First, the cross-sectional associations between functional health literacy, cognitive ability, and self-reported diabetes were investigated. Second, participants without diabetes at baseline were followed-up for up to 10 years to determine whether functional health literacy and cognitive ability were independently associated with newly reporting diabetes during follow-up.

METHODS

Participants

For peer review only

This study used data from core members of the ELSA study, a prospective cohort study of community-dwelling adults residing in England. ELSA was designed to be representative of adults aged 50 years and older living in England.[25] The wave 1 (2002-03) sample consisted of 11,391 participants who had previously participated in the Health Survey for England between 1998 and 2001, who were born before 1 March 1952, and who were living in a private household in England.[25] ELSA participants have been followed up every two years and the sample has been refreshed at waves 3, 4, 6 and 7 to ensure the sample is representative of adults aged over 50 years. The present study used data from waves 2 (2004-05) to 7 (2014-15), and baseline, here, was considered to be wave 2 (n=8,726), which was when the functional health literacy assessment was introduced.

At each wave, a face-to-face interview was used to measure topics including health, lifestyle and economic circumstances. Face-to-face interviews were carried out in the participant's own home using computer-assisted interviewing. Participants answered a self-completion questionnaire including questions about diet and alcohol consumption. A nurse interview was carried out at waves 2, 4 and 6 to assess physical measurements including height and weight, and blood and saliva samples were taken to measure biomarkers of disease. Detailed descriptions of the sample design and data collected in ELSA are reported elsewhere.[25]

Ethical approval was obtained from the NHS Multicentre Research Ethics Committee, London (reference: MREC/01/2/91). Written informed consent was obtained from all ELSA participants. This study conformed to the principles embodied in the Declaration of Helsinki.

Patient and Public Involvement

Participants were not involved in the development of any part of this study.

Measures

Diabetes

For peer review only

Baseline diabetes status: Individuals who answered “yes” to “Has a doctor ever told you that you have diabetes?” at wave 2 were categorised as having diabetes. This question did not differentiate which type of diabetes the participant was diagnosed with.

Diabetes during follow-up: This analysis was restricted to participants who did not self-report diabetes at wave 2 and who had at least one wave of follow-up between waves 3 and 7. Participants who did not self-report diabetes at wave 2 and who subsequently answered “yes” to “Has a doctor ever told you that you have diabetes?” any time between waves 3 and 7 were categorised as having newly diagnosed diabetes during follow-up. As all participants were aged over 50 years at diagnosis, these cases are probably cases of type 2 diabetes.

Date of diabetes diagnosis: Individuals who self-reported diabetes were asked which month and year they were diagnosed. Date of diabetes diagnosis was used to calculate the time between wave 2 assessment and diabetes diagnosis.

Functional health literacy

A 4-item functional health literacy test taken from the Adult Literacy and Life Skills Survey,[26] and the International Adult Literacy Survey[27] was administered during the wave 2 interview. This test assessed health-related reading comprehension skills which are thought to be required to successfully understand written materials commonly encountered in healthcare. Participants were presented with a piece of paper containing a label for a packet of over-the-counter medication. Participants were asked four questions about the information on this label (e.g., “what is the maximum number of days you may take this medicine?”). The score was the number of correctly answered questions. As has been done in other studies,[28, 29] performance was categorised as adequate (4/4 correct) or limited (<4 correct).

Cognitive ability

For peer review only - http://bmjopen.bmj.com/site/about/guidelines.xhtml

Scores on different cognitive tests tend to be positively correlated.[30] Data reduction techniques such as principal component analysis (PCA) are often used to capture the covariance among a range of difference cognitive tests. This shared variance can then be used as a measure of general cognitive ability.[31] Four tests administered during the wave 2 interview that are designed to assess cognitive domains that decline with increasing age[32] were entered into a PCA to create a measure general cognitive ability.

Word list learning tests, in which participants are required to remember a list of words immediately and then after a delay are commonly used to assess verbal declarative memory and learning.[33] Here, the immediate and delayed word recall tests were used. Participants were read a list of 10 words and were asked to immediately recall as many of the words as possible. The score was the number of words recalled immediately. After a short delay, in which the words were not repeated, participants were asked to remember the 10 words again. The score was the number of words recalled after a delay. Verbal fluency tests, in which participants are asked to produce as many words as possible in a set time following a set of rules, are often used to measure executive function.[33] Category fluency was used to assess executive function in ELSA. Participants were instructed to name as many animals as possible. The score was the number of animals named in 60 seconds. Tests of processing speed involve completing a simple task as quickly as possible and common tests include using a code to write as many symbols as possible, or finding symbols amongst distractors and scoring them out as quickly as possible.[33, 34] Letter cancellation was used to assess processing speed. Participants were presented with a piece of paper containing letters of the alphabet arranged in rows and columns. The task was to scan the piece of paper and score out all Ps and Ws. The score was the combined number of Ps and Ws scored out in 60 seconds.

Scores of 0 on animal fluency (n=48) and letter cancellation (n=3) were removed as scores of 0 on these tests suggest participants either did not complete the task or did not understand the

For peer review only - http://bmjopen.bmj.com/site/about/guidelines.xhtml

task. Scores of ≥50 on animal fluency (n=4), and ≥60 on the letter cancellation (n=3) were removed as these scores were extremely high given the 60 second time limit for these tests and these values are greater than 4 SDs from the mean.

We did not include tests of self-reported memory, prospective memory or orientation in time in the measure of general cognitive ability. Self-reported memory was not included because this is a subjective test. Prospective memory was not included because the test consists of only one trial. Orientation in time is a four item test in which participants are asked to recall the date. It has limited variance and is most frequently used as a brief screening tool for cognitive impairment.

Only the first principal component had an eigenvalue >1. The scree plot also indicated one component. Scores from the first principal component were saved and used as a measure of cognitive ability (mean=0.00, SD=1.00). The first component accounted for 57% of the variance in the scores on the four cognitive tests. The loadings were: Immediate word recall=0.83, delayed word recall=0.85, animal fluency=0.72, and letter cancellation=0.58.

Covariates

Age (in years), sex, BMI, health behaviours, number of cardiovascular comorbidities, and indicators of socioeconomic status were used as covariates. Unless otherwise stated, all were self-reported at the wave 2 interview. Prior to releasing data, ELSA set the age of all participants aged over 90 years to 90 years to reduce the risk of disclosure. Participants were asked whether they smoked cigarettes nowadays and were categorised as current smokers or non-smokers. Participants were asked how often they took part in moderate and vigorous physical activity (more than once a week, once a week, one to three times a month, and hardly ever/never). Physical activity levels were categorised as vigorous activity at least once per week, moderate activity at least once per week, and physically inactive. Participants were

For peer review only - http://bmjopen.bmj.com/site/about/guidelines.xhtml

asked about their frequency of alcohol consumption in the past 12 months in the self-completion questionnaire. This was categorised as never, rarely, at least once a month, at least once a week, and daily/almost daily. Height and weight, measured during the wave 2 nurse interview, were used to calculate BMI (kg/m^2). Cardiovascular comorbidities were assessed by counting the number of self-reported cardiovascular conditions from hypertension, angina, heart attack, heart murmur, abnormal heart rhythm, stroke, and high cholesterol. Age that participants left full-time education was categorised as: age 14 or under, 15-16 years, 17-18 years, and age 19 or older. Social class was categorised using the National Statistics Socioeconomic Classification 3 categories;[35] managerial and professional, intermediate, and routine and manual.

Analysis

All analyses was performed in R. Independent t-tests were used to compare those with and without diabetes at wave 2 and those who did and did not self-report diabetes at follow-up on normally-distributed continuous variables. Mann-Whitney U tests were used for non-normal continuous variables, and Chi-squared tests were used for categorical variables. Spearman rank-order correlations were calculated between all predictor variables and co-variables.

Binary logistic regression was used to test the cross-sectional association of functional health literacy and cognitive ability with diabetes reported at wave 2. Cox regression was used to investigate whether functional health literacy and cognitive ability test scores at wave 2 were associated with newly reported diabetes between waves 2 and 7. In the Cox regression analysis, time-to-event was taken as the difference, in days, between date of wave 2 interview and date of diabetes diagnosis for those who self-reported diabetes. For other participants, time-to-event was the difference between date of wave 2 interview and the date of last interview. Month and

For peer review only - http://bmjopen.bmj.com/site/about/guidelines.xhtml

year, but not day, were recorded for date of interview and date of diabetes diagnosis. To create a date variable (yyyy.mm.dd), the day was set to the middle of the month.

For the logistic regressions and Cox regressions, 7 models were run. Age and sex were entered into all models. Functional health literacy and cognitive ability were entered individually in models 1 and 2, respectively. Both functional health literacy and cognitive ability were added in Model 3 to determine whether the size of the functional health literacy-diabetes and cognitive ability-diabetes associations changed when simultaneously entering both these variables. Functional health literacy and cognitive ability were also entered together in models 4-7. To assess whether BMI and health behaviours accounted for these associations, BMI, smoking status, alcohol consumption, and physical activity were added in Model 4. Diabetes is a risk factor for cardiovascular disease.[36] Associations between poorer cognitive ability and cardiovascular disease are also well established.[37, 38] It is possible that any association between functional health literacy and cognitive ability with diabetes may be because of these associations with cardiovascular disease. To determine whether any association between functional health literacy and cognitive ability with diabetes was attenuated when adjusting for cardiovascular disease, number of cardiovascular comorbidities was added in Model 5. Age of leaving full-time education and occupational social class were added in Model 6. A fully-adjusted model (Model 7) adjusted for functional health literacy, cognitive ability, and all covariates.

This study was interested in the associations of functional health literacy and cognitive ability with self-reported diabetes and the independence of these associations with respect to other health and socioeconomic-related variables. In the main text we report the odd ratios (ORs) and hazard ratios (HRs) for functional health literacy and cognitive ability only. The estimates for all variables entered into the models are reported in the Supplementary materials.

For peer review only - http://bmjopen.bmj.com/site/about/guidelines.xhtml

RESULTS

Of the 8,726 ELSA participants who completed wave 2, 3 participants were removed who answered "don't know" to whether a doctor had diagnosed them with diabetes. A further 54 participants were removed because they selected that they had "diabetes or high blood sugar" from a Showcard listing cardiovascular conditions, but when asked whether a doctor had ever told them they had diabetes, they answered "no". The analytic sample consisted of 8,669 participants. Participant characteristics are reported in Table 1.

Baseline diabetes status

At baseline, 708 (8.2%) participants self-reported a diagnosis of diabetes. Compared to those without diabetes, those with diabetes were more likely to have limited functional health literacy (42.2% versus 32.3%) and have lower cognitive ability (diabetes mean=-0.36, SD=0.97; no diabetes mean=0.03, SD=1.00; Cohen's d=0.40). Participants with diabetes were older (diabetes mean=69.36, SD=9.16; no diabetes mean=66.46, SD=9.70) and more likely to be male (53.5% versus 44.2%) than those without. Those with diabetes were also more likely to leave full-time education at a younger age, be from a less professional social class, have a higher BMI, consume less alcohol, be inactive, and self-report more cardiovascular comorbidities (Table 1). Rank-order correlations between predictor variables and co-variables are reported in Table 2. Adequate functional health literacy was moderately correlated with higher cognitive ability (rho=0.31, p<0.001).

ORs and 95% confidence intervals (CIs) for the associations between functional health literacy and cognitive ability with self-reported diabetes at wave 2 are reported in Table 3 (and Supplementary Table S1). Box-Tidwell tests were performed whereby an interaction term between each continuous predictor variable and the log of that variable were added to the model to check the assumption that there is a linear relationship between each continuous

For peer review only - http://bmjopen.bmj.com/site/about/guidelines.xhtml

predictor and the logit of the outcome. The interaction between age and log(age) and the interaction between number of cardiovascular comorbidities and log(number of cardiovascular comorbidities) was significant. Therefore the assumptions of the linearity of the logit was violated. To overcome this, an age-squared term was included in all models, and a squared term for number of cardiovascular comorbidities was included in Models 5 and 7.

Participants with adequate functional health literacy were 29% less likely to self-report diabetes (Model 1 OR=0.71; 95% CI=0.61 to 0.84). A 1 SD higher cognitive ability was associated with 27% lower odds of self-reported diabetes (Model 2 OR=0.73; 95% CI=0.67 to 0.80). The association between functional health literacy and diabetes was attenuated by 38% (OR=0.82; 95% CI=0.69 to 0.98) and the association between cognitive ability and diabetes was attenuated by 19% (OR=0.78; 95% CI=0.70 to 0.86) when entering both functional health literacy and cognitive ability in Model 3. Both remained significantly associated with diabetes.

BMI and health behaviours were added in Model 4. The associations between functional health literacy and cognitive ability with diabetes were attenuated and no longer significant. The cognitive ability-diabetes association was not attenuated after adjusting for cardiovascular comorbidities (Model 5) or when adjusting for education and social class (Model 6). Cognitive ability remained significantly associated with diabetes in these models. The association between functional health literacy and diabetes was slightly attenuated and no longer significant when adjusting for cardiovascular comorbidities (Model 5) and education and social class (Model 6). In the fully adjusted model (Model 7), the size of the associations between functional health literacy and cognitive ability with diabetes were reduced further and were non-significant.

In the fully-adjusted model (Model 7; Supplementary Table S1) older age, male sex, having a higher BMI, and reporting more cardiovascular comorbidities were associated with higher odds

For peer review only - http://bmjopen.bmj.com/site/about/guidelines.xhtml

of having diabetes. The association between number of cardiovascular comorbidities and diabetes became less strong as the number of comorbidities increased. Those who reported drinking alcohol at least once per month, rarely, or who never drank alcohol in the last 12 months were more likely to self-report diabetes when compared to those who reported drinking daily/almost daily. Compared to those who reported being physically inactive, those who took part in moderate or vigorous physical activity at least once per week were less likely to self-report diabetes.

Diabetes during follow-up

Of the 7,961 participants who did not self-report diabetes at wave 2, 6,961 participants had at least one wave of follow-up between waves 3 and 7. They form the analytic sample for the association between functional health literacy, cognitive ability and self-reported diabetes during follow-up. A total of 506 (7.3%) participants reported a new diagnosis of diabetes between wave 3 and wave 7, whereas 6,455 (92.7%) participants did not. Median time to follow-up was 9.5 years. Mean time to censor was 4.7 years (SD=3.1) for those with diabetes and 7.8 years (SD=2.9) for those without. Participant characteristics are reported in Table 1. Compared to participants who did not self-report diabetes during follow-up, those who did were more likely to have limited functional health literacy (38.8% versus 30.3%) and had lower cognitive ability (diabetes mean=-0.04, SD=0.89; no diabetes mean=0.10, SD=0.98, Cohen's d=0.15) at wave 2. Participants who reported diabetes were younger (diabetes mean=65.51, SD=8.59; no diabetes mean=66.0; SD=9.53) and more likely to be male (51.8% versus 43.2%) than those without. Compared to those without diabetes, participants who reported diabetes during follow-up were more likely to have left full-time education at a younger age, be from a less professional social class, smoke, consume less alcohol, be inactive, and to report more cardiovascular comorbidities at wave 2 (Table 1).

For peer review only - http://bmjopen.bmj.com/site/about/guidelines.xhtml

The HRs and 95% CIs for the association between functional health literacy, cognitive ability and self-reporting diabetes during follow-up are reported in Table 4 (and Supplementary Table S2). Adequate functional health literacy at wave 2 was associated with a 36% lower risk of reporting diabetes (Model 1 HR=0.64; 95% CI=0.53 to 0.77). A 1 SD higher cognitive ability at wave 2 was associated with a 23% lower risk of reporting diabetes (Model 2 HR=0.77; 95% CI=0.69 to 0.85). The association between functional health literacy and diabetes was attenuated by 22% after adjustment for cognitive ability (Model 3 HR=0.72; 95% CI=0.59 to 0.87), and the association between cognitive ability and diabetes was attenuated by 9% after adjusting for functional health literacy (HR=0.79; 95% CI=0.71 to 0.88). Both functional health literacy and cognitive ability remained significant predictors of reporting diabetes during follow-up.

BMI and health behaviours were added in Model 4. The associations of functional health literacy and cognitive ability with reporting diabetes were further attenuated but remained statistically significant. When adjusting for number of cardiovascular comorbidities, the association between functional health literacy and cognitive ability with diabetes remained almost unchanged (Model 5) and both remained significantly associated with diabetes. Education and social class was added in Model 6. The size of the association between functional health literacy and cognitive ability with diabetes were slightly reduced but remained statistically significant. In the fully-adjusted model (Model 7) the associations between functional health literacy and cognitive ability and reporting diabetes were further reduced and no longer significant.

In the fully-adjusted model (Model 7; Supplementary Table S2) male participants, those with a higher BMI, current smokers, and those who reported consuming alcohol rarely (compared to daily/almost daily) at wave 2 were more likely to report diabetes during follow-up. Participants

For peer review only - http://bmjopen.bmj.com/site/about/guidelines.xhtml

who reported leaving education at age 19 years or older were less likely to report diabetes during follow-up compared to those who left at age 14 years or younger.

Sensitivity analysis

Missing data

There was missing data. For the cross-sectional analyses, 70% of participants had complete data. For the longitudinal analyses, 75% of participants had complete data. All models were re-run using only participants with complete data on all variables. These results are reported in Supplementary Tables S3 and S4. The pattern of associations were generally similar; however, the sizes of the associations tended to be slightly weaker compared to the full sample. For the cross-sectional analysis, functional health literacy was no longer significantly associated with diabetes status in Model 3 when adjusting for functional health literacy and cognitive ability (Supplementary Table S3). For the longitudinal analysis, when adjusting for BMI and health behaviours (Model 4; Supplementary Table S4), functional health literacy was no longer associated with reporting diabetes during follow-up.

Undiagnosed diabetes

It is possible that some participants not reporting diabetes may have undiagnosed diabetes. To identify participants who may have undiagnosed diabetes HbA_{1c} levels collected by blood draw during the nurse interview (waves 2, 4, and 6) were used.[25] Participants who did not report diabetes but who had HbA_{1c} levels of $\geq$47.5 mmol/mol (6.5%) were categorised as having suspected undiagnosed diabetes. The models were re-run after removing these individuals to determine whether the results differ from those reported in the main models.

A total of 5,783 participants who formed the analytical sample for the cross-sectional analysis had HbA_{1c} levels available from the wave 2 nurse interview (399 self-reporting diabetes; 5,384 not self-reporting diabetes). Of the 5,384 participants who did not self-report diabetes at wave

For peer review only - http://bmjopen.bmj.com/site/about/guidelines.xhtml

2 and who had HbA_{1c} levels available at wave 2, 112 (2.1%) participants had HbA_{1c} levels of ≥47.5 mmol/mol (6.5%). Models were re-run on this sub-sample after removal of these 112 participants with suspected undiagnosed diabetes (n=5,671). The results are reported in Supplementary Table S5. The associations between cognitive ability and diabetes status at wave 2 are very similar to those reported in the main model. Using this sub-sample, the size of the associations between functional health literacy and diabetes were reduced and were no longer significant in Model 1 (functional health literacy only; Supplementary Table S5) and Model 3 (functional health literacy and cognitive ability; Supplementary Table S5).

The Cox regressions were also re-run after removal of participants with suspected undiagnosed diabetes. The follow-up period was restricted to waves 3 to 6 (mean follow-up=7.5 years), as HbA_{1c} levels were not available at wave 7. A total of 4,425 participants who formed the analytical sample for the Cox models had HbA_{1c} levels collected at wave 4 and/or wave 6 (279 self-reporting diabetes between waves 3 and 6; 4,146 not self-reporting diabetes during follow-up). 147 participants who reported not having diabetes at waves 3 and 4 had HbA_{1c} levels of ≥47.5 mmol/mol (6.5%) at wave 4 and were removed. A further 72 participants reported not having diabetes between waves 3 and 6 but had HbA_{1c} levels of ≥47.5 mmol/mol (6.5%) at wave 6 and were removed. The Cox regression models were re-run on this sample (n=4,206; 212 reporting diabetes during follow-up; 3,994 not reporting diabetes during follow-up). The results are reported in Supplementary Table S6. The size of the associations between limited functional health literacy and self-reporting diabetes during follow-up became even stronger. In the fully-adjusted model (Model 7, Supplementary Table S6), the association between limited functional health literacy and diabetes remained significant. For cognitive ability, the strength of the associations were generally similar to the main models. However, after adjusting for BMI and health behaviours (Model 4, Supplementary Table S6) the size of the association between cognitive ability and diabetes was slightly attenuated and no longer significant.

For peer review only - http://bmjopen.bmj.com/site/about/guidelines.xhtml

DISCUSSION

Using a sample of middle-aged and older adults living in England, the present study found that adequate functional health literacy and better cognitive ability were associated with lower odds of self-reporting diabetes. These associations were attenuated when functional health literacy and cognitive ability were entered in the same model, though both independently contributed to diabetes. These associations were further attenuated and non-significant when adjusting for BMI and health behaviours. Adjusting for cardiovascular comorbidities and indicators of socioeconomic status did not attenuate the association between cognitive ability and diabetes, however, for functional health literacy there was a small attenuation and these associations were no longer significant. When adjusting for all covariates simultaneously, neither functional health literacy nor cognitive ability was associated with diabetes at wave 2.

Adequate health literacy and better cognitive ability, measured at wave 2, were associated with reduced risk of self-reporting diabetes during a median of 9.5 years follow-up. Both functional health literacy and cognitive ability were independently associated with self-reported diabetes when both were entered in the same model. These associations remained when separately adjusting for BMI and health behaviours, cardiovascular comorbidities, and education and social class. However, neither health literacy nor cognitive ability were associated with reporting diabetes during follow-up when all covariates were entered together.

Previous cross-sectional studies have found that individuals with lower functional health literacy are more likely to report having diabetes[11, 12] and longitudinal studies have found that that lower cognitive ability earlier in life is associated with an increased risk of diabetes.[5, 6] The present study is the first longitudinal study to examine whether functional health literacy is associated with self-reporting a new diagnosis of diabetes, and the first to examine whether cognitive ability and functional health literacy have independent associations with

For peer review only - http://bmjopen.bmj.com/site/about/guidelines.xhtml

diabetes. The results reported here suggest that cognitive capabilities and health-related reading comprehension skills, though related, contribute independently to diabetes.

There are obvious similarities between tests of cognitive ability and functional health literacy. The Rapid Estimate of Adult Literacy in Medicine (REALM)[39] is a popular health literacy test which involves the ability to read and pronounce health-related words of varying complexity. More ecologically valid assessments of functional health literacy such as the Test of Functional Health Literacy in Adults (TOFHLA)[10] and the health literacy test used in the current study involve participants using mock health-related props, such as prescription labels or a medical appointment slips, and answering questions about the information presented. Successful completion of these tests will require the ability to process information, plan and problem solve (i.e.,. cognitive ability).[2]

Some have suggested that functional health literacy variance is mostly overlapping with cognitive ability.[23, 40] If this were true, one would expect the association between functional health literacy and diabetes to be fully attenuated when adjusting for cognitive ability. This is not what was found here. Only some of the association of functional health literacy and diabetes was accounted for by cognitive ability. The level of independence between health literacy and cognitive ability may vary depending on the assessments used to measure health literacy and cognitive ability.[22] The cognitive ability measure used here included four brief cognitive ability tests that assessed memory, executive function and processing speed, and did not include other important domains of cognitive function, such as reasoning, that are known to load highly on general cognitive ability.[41] The health literacy assessment was also very brief. Some of the unique contribution of functional health literacy might be residual cognitive capability that was not picked up by the relatively brief measures of cognitive ability used here.[42] However, unique associations of health literacy and cognitive ability with health have been reported when using a variety of different functional health literacy tests, including the

For peer review only - http://bmjopen.bmj.com/site/about/guidelines.xhtml

REALM[23], the TOFHLA[21, 23] and the ELSA health literacy test[22]. Though attenuated, functional health literacy has also been found to have had unique associations with health after adjusting for cognitive ability created using a comprehensive test battery consisting of well-validated cognitive tests[23]. Therefore, low health literacy and poorer cognitive ability may contribute unique disadvantages in terms of navigating healthcare and looking after one's own health.[22]

This study was also interested in examining whether functional health literacy and cognitive ability were associated with reporting diabetes independent of other health-related and socioeconomic risk factors for diabetes. The largest attenuation was seen when entering health behaviours and BMI into the models. BMI and health behaviours fully attenuated the relationship between functional health literacy, cognitive ability and reporting diabetes at baseline, and partly attenuated the relationship between functional health literacy, cognitive ability and reporting diabetes during follow-up. Better cognitive ability has been associated with health promoting behaviours such as following a healthy diet and taking part in regular exercise.[4, 43-45] Whereas some studies have found associations between better functional health literacy and taking part in health promoting behaviours,[46] others have not.[47] Individuals with higher functional health literacy and cognitive ability might be better equipped with the health-related skills and knowledge, and the general cognitive capabilities needed to take better care of themselves[2, 48] and to follow health advise such as eating well and exercising, which, in turn, could reduce the risk of developing diabetes.[1]

Education also partly attenuated the association between functional health literacy and cognitive ability with reporting diabetes during follow-up. The association between better functional health literacy and cognitive ability with higher levels of education are well established.[7, 49] Education may lead to better cognitive ability and functional health literacy, which in turn may lead to better health-related skills and lower rates of diabetes.[23] Higher

For peer review only - http://bmjopen.bmj.com/site/about/guidelines.xhtml

cognitive ability in early life has been found to predict later educational attainment.[49] An alternative but not mutually exclusive explanation could be that higher cognitive ability may equip an individual with the skills needed to obtain higher educational qualifications. Higher educational attainment, in turn, may lead to better health (and lower risk of diabetes) by, for example, increasing health-related knowledge and decision-making skills.[23] In the current study, social class was not found to have associations with diabetes and did not appear to play an attenuating role in the association between health literacy and cognitive ability with diabetes.

This study has a number of strengths and limitations. A key strength is that it examined the association of functional health literacy, cognitive ability and reporting diabetes longitudinally. Another strength is the relatively large sample size. One limitation is that only a sub-sample of participants had complete data. Those with missing data may be those with the lowest functional health literacy and cognitive ability scores. ELSA may also suffer from selective attrition such that those with increased risk of developing diabetes may be less likely to return for follow-up. The results reported here may not generalise to those with the lowest functional health literacy and/or cognitive ability. The rates of diabetes reported here do not fully match those reported in national statistics. Compared to the 2004/05 National Diabetes Audit for England and Wales, rates of diabetes in the current study were lower for those aged 55 to 69 years (this study: 8.4% in men and 5.6% in women; National Diabetes Audit: approximately 10% in men, and 7% in women), but comparable in those aged 70 to 84 years (this study: 13.6% in men and 9.9% in women; National Diabetes Audit: approximately 13.5% in men and 10% in women).[50] Therefore the current sample is not fully representative of people with diabetes living in England.

Another limitation is that diabetes status was self-reported. As has been shown in other ELSA studies, there is a relatively high rate of agreement between self-reported diabetes and fasting

For peer review only - http://bmjopen.bmj.com/site/about/guidelines.xhtml

blood glucose in ELSA; however, 1.7% of participants had undiagnosed diabetes.[51] Sensitivity analysis was performed in the current study to try to identify and remove individuals with undiagnosed diabetes. Although the results were generally similar after removal of those with suspected undiagnosed diabetes, we found that health literacy was no longer associated with cross-sectional diabetes status in the sub-sample of participants with HbA_{1c} levels. It is not clear whether these differences are due to removal of participants with suspected undiagnosed diabetes, or if it was due to bias caused by using a smaller sub-sample of participants who also attended the nurse interview and provided a blood sample.

The functional health literacy test used here was a brief, four-item test which had limited variance (67% of participants scored the highest score) and the psychometric properties of this measure are unknown. Although brief, this test was sensitive enough to have associations with self-reported diabetes during follow-up, and it has previously been found to have associations with mortality.[22] This brief measure only assessed functional health literacy and did not measure other components of health literacy.[8] More detailed, self-report measures of health literacy are available that assess a range of other health literacy skills, including the (self-reported) ability to access, appraise and apply health information.[52] An important next step would be to test the associations between health literacy and cognitive ability with diabetes using more detailed tests of health literacy that cover a range of other health literacy skills in addition to health-related reading comprehension.

This study found that adequate functional health literacy and higher cognitive ability were independently associated with lower odds of self-reporting diabetes at wave 2 and with reduced rates of self-reporting a new diagnosis of diabetes during a median of 9.5 years follow-up. Individuals with poor functional health literacy and/or cognitive ability might lack the health-related reading and writing skills and the general cognitive capabilities required to look after

For peer review only

their health throughout life, which in turn, may increase the risk of being diagnosed with diabetes.

For peer review only

For peer review only - http://bmjopen.bmj.com/site/about/guidelines.xhtml

Funding This work was supported by the University of Edinburgh Centre for Cognitive Ageing and Cognitive Epidemiology, part of the cross council Lifelong Health and Wellbeing Initiative, funded by the Biotechnology and Biological Sciences Research Council (BBSRC), and Medical Research Council (MRC) (grant number MR/K026992/1).

Conflict of Interest No potential conflicts of interest were reported.

Author Contributions Chloe Fawns-Ritchie contributed to the conception and design of the project, analysed the data, interpreted the data, drafted the initial manuscript, and critically revised the manuscript. Jackie Price contributed to the conception and design of the project, interpreted the data, and critically revised the manuscript. Ian J. Deary contributed to the conception and design of the project, interpreted the data, and critically revised the manuscript.

Acknowledgements We thank our late colleague, John Starr, for his contribution to the conception and design of this study. The authors thank the participants of the ELSA study. We are grateful to the UK Data service for supplying the data.

Data accessibility Anonymised data from the English Longitudinal Study of Ageing is available from the UK Data Service (https://https://www.ukdataservice.ac.uk/).

For peer review only

REFERENCES

1 Hussain A, Claussen B, Ramachandran A*, et al.* Prevention of type 2 diabetes: a review. *Diabetes Res Clin Pract* 2007;76:317-26.

2 Gottfredson LS. Intelligence: is it the epidemiologists' elusive "fundamental cause" of social class inequalities in health? *J Pers Soc Psychol* 2004;86:174-99.

3 Malanchini M, Rimfeld K, Allegrini AG*, et al.* Cognitive ability and education: How behavioural genetic research has advanced our knowledge and understanding of their association. *Neuroscience & Biobehavioral Reviews* 2020;111:229-45.

4 Batty GD, Deary IJ, Macintyre S. Childhood IQ in relation to risk factors for premature mortality in middle-aged persons: the Aberdeen Children of the 1950s study. *J Epidemiol Community Health* 2007;61:241-7.

5 Mõttus R, Luciano M, Starr JM*, et al.* Diabetes and life-long cognitive ability. *J Psychosom Res* 2013;75:275-8.

6 Twig G, Gluzman I, Tirosh A*, et al.* Cognitive function and the risk for diabetes among young men. *Diabetes care* 2014;37:2982-8.

7 Nielsen-Bohlman L, Panzer AM, Kindig DA. *Health Literacy: A Prescription to End Confusion*. Washinton, DC: The National Academies Press 2004.

8 Sørensen K, Van Den Broucke S, Fullam J*, et al.* Health literacy and public health: A systematic review and integration of definitions and models. *BMC Public Health* 2012;12:80-.

9 Nutbeam D. The evolving concept of health literacy. *Social Science & Medicine* 2008;67:2072-8.

10 Parker RM, Baker DW, Williams MV*, et al.* The test of functional health literacy in adults: A new instrument for measuring patients' literacy skills. *Journal of General Internal Medicine* 1995;10:537-41.

For peer review only - http://bmjopen.bmj.com/site/about/guidelines.xhtml

11 Adams RJ, Appleton SL, Hill CL*, et al.* Risks associated with low functional health literacy in an Australian population. *Med J Aust* 2009;191:530-4.

12 Wolf MS, Gazmararian JA, Baker DW. Health literacy and functional health status among older adults. *Arch Intern Med* 2005;165:1946-52.

13 Quartuccio M, Simonsick EM, Langan S*, et al.* The relationship of health literacy to diabetes status differs by sex in older adults. *Journal of Diabetes and its Complications* 2018;32:368-72.

14 Caruso R, Magon A, Baroni I*, et al.* Health literacy in type 2 diabetes patients: a systematic review of systematic reviews. *Acta Diabetol* 2018;55:1-12.

15 Marciano L, Camerini AL, Schulz PJ. The Role of Health Literacy in Diabetes Knowledge, Self-Care, and Glycemic Control: a Meta-analysis. *J Gen Intern Med* 2019;34:1007-17.

16 Al Sayah F, Majumdar SR, Williams B*, et al.* Health literacy and health outcomes in diabetes: a systematic review. *J Gen Intern Med* 2013;28:444-52.

17 Bennett JS, Boyle PA, James BD*, et al.* Correlates of health and financial literacy in older adults without dementia. *BMC Geriatrics* 2012;12:30.

18 Murray C, Johnson W, Wolf MS*, et al.* The Association between Cognitive Ability across the Lifespan and Health Literacy in Old Age: The Lothian Birth Cohort 1936. *Intelligence* 2011;39:178-87.

19 Wolf MS, Curtis LM, Wilson EA*, et al.* Literacy, cognitive function, and health: results of the LitCog study. *General Internal Medicine* 2012;27:1300-7.

20 Lamar M, Wilson RS, Yu L*, et al.* Associations of literacy with diabetes indicators in older adults. *J Epidemiol Community Health* 2019;73:250-5.

21 Baker DW, Wolf MS, Feinglass J*, et al.* Health literacy, cognitive abilities, and mortality among elderly persons. *J Gen Intern Med* 2008;23:723-6.

For peer review only - http://bmjopen.bmj.com/site/about/guidelines.xhtml

22 Bostock S, Steptoe A. Association between low functional health literacy and mortality in older adults: longitudinal cohort study. *BMJ* 2012;344:e1602.

23 Mõttus R, Johnson W, Murray C*, et al.* Towards understanding the links between health literacy and physical health. *Health Psychol* 2014;33:164-73.

24 Fawns-Ritchie C, Starr JM, Deary IJ. Role of cognitive ability in the association between functional health literacy and mortality in the Lothian Birth Cohort 1936: a prospective cohort study. *BMJ Open* 2018;8:e022502.

25 Steptoe A, Breeze E, Banks J*, et al.* Cohort profile: the English Longitudinal Study of Ageing. *Int J Epidemiol* 2013;42:1640-8.

26 Desjardins R, Murray S, Clermont Y*, et al.* Learning a living: First results of the adult literacy and life skills survey. *Statistics Canada catalogue* 2005.

27 Kirsch IS. The International Adult Literacy Survey (IALS): Understanding What is Measured. 2001;2001:i-61.

28 Kobayashi LC, Wardle J, von Wagner C. Limited health literacy is a barrier to colorectal cancer screening in England: evidence from the English Longitudinal Study of Ageing. *Prev Med* 2014;61:100-5.

29 Gale CR, Deary IJ, Wardle J*, et al.* Cognitive ability and personality as predictors of participation in a national colorectal cancer screening programme: the English Longitudinal Study of Ageing. *J Epidemiol Community Health* 2015;69:530-5.

30 Spearman C. "General Intelligence", objectively determined and measured. *The American Journal of Psychology* 1904;15:201-92.

31 Salthouse T. *Major issues in cognitive aging*: Oxford University Press 2010.

32 Salthouse TA. Selective review of cognitive aging. *Journal of the International Neuropsychological Society* 2010;16:754-60.

For peer review only - http://bmjopen.bmj.com/site/about/guidelines.xhtml

33 Lara J, Cooper R, Nissan J*, et al.* A proposed panel of biomarkers of healthy ageing. *BMC Medicine* 2015;13:222.

34 Lezak MD, Howieson DB, Bigler ED*, et al. Neuropsychological Assessment*. Oxford: Oxford University Press 2012.

35 Rose D, Pevalin DJ, O'Reilly K. *The National Statistics Socio-economic Classification: origins, development and use*: Palgrave Macmillan Basingstoke 2005.

36 Sarwar N, Gao P, Seshasai SR*, et al.* Diabetes mellitus, fasting blood glucose concentration, and risk of vascular disease: a collaborative meta-analysis of 102 prospective studies. *Lancet* 2010;375:2215-22.

37 Rostamian S, Mahinrad S, Stijnen T*, et al.* Cognitive impairment and risk of stroke: a systematic review and meta-analysis of prospective cohort studies. *Stroke* 2014;45:1342-8.

38 Hart CL, Taylor MD, Smith GD*, et al.* Childhood IQ and cardiovascular disease in adulthood: prospective observational study linking the Scottish Mental Survey 1932 and the Midspan studies. *Social Science & Medicine* 2004;59:2131-8.

39 Davis TC, Long SW, Jackson RH*, et al.* Rapid estimate of adult literacy in medicine: a shortened screening instrument. *Family Medicine* 1993;25:391-5.

40 Reeve CL, Basalik D. Is health literacy an example of construct proliferation? A conceptual and empirical evaluation of its redundancy with general cognitive ability. *Intelligence* 2014;44:93-102.

41 Salthouse TA. Localizing age-related individual differences in a hierarchical structure. *Intelligence* 2004;32:541-61.

42 Fawns-Ritchie C, Starr JM, Deary IJ. Health literacy, cognitive ability and smoking: a cross-sectional analysis of the English Longitudinal Study of Ageing. *BMJ Open* 2018;8:e023929.

For peer review only - http://bmjopen.bmj.com/site/about/guidelines.xhtml

43 Batty GD, Deary IJ, Schoon I, *et al.* Childhood mental ability in relation to food intake and physical activity in adulthood: the 1970 British Cohort Study. *Pediatrics* 2007;119:e38-45.

44 Wraw C, Der G, Gale CR, *et al.* Intelligence in youth and health behaviours in middle age. *Intelligence* 2018;69:71-86.

45 Batty GD, Deary IJ, Schoon I, *et al.* Mental ability across childhood in relation to risk factors for premature mortality in adult life: the 1970 British Cohort Study. *J Epidemiol Community Health* 2007;61:997-1003.

46 von Wagner C, Knight K, Steptoe A, *et al.* Functional health literacy and health-promoting behaviour in a national sample of British adults. *J Epidemiol Community Health* 2007;61:1086-90.

47 Wolf MS, Gazmararian JA, Baker DW. Health literacy and health risk behaviors among older adults. *Am J Prev Med* 2007;32:19-24.

48 Deary IJ, Weiss A, Batty GD. Intelligence and Personality as Predictors of Illness and Death: How Researchers in Differential Psychology and Chronic Disease Epidemiology Are Collaborating to Understand and Address Health Inequalities. *Psychol Sci Public Interest* 2010;11:53-79.

49 Deary IJ, Strand S, Smith P, *et al.* Intelligence and educational achievement. *Intelligence* 2007;35:13-21.

50 The Information Centre. National Diabetes Audit: Key findings about the quality of care for people with diabetes in England, incorporating registrations from Wales. Report for the audit period 2004/05. 2006.

51 Pierce MB, Zaninotto P, Steel N, *et al.* Undiagnosed diabetes-data from the English longitudinal study of ageing. *Diabetic Med* 2009;26:679-85.

For peer review only

For peer review only - http://bmjopen.bmj.com/site/about/guidelines.xhtml

52 Sørensen K, Pelikan JM, Rothlin F*, et al.* Health literacy in Europe: comparative results of the European health literacy survey (HLS-EU). *European Journal of Public Health* 2015;25:1053-8.

For peer review only

For peer review only - http://bmjopen.bmj.com/site/about/guidelines.xhtml

Table 1 Participant characteristics by diabetes status

	Diabetes reported at wave 2				Diabetes reported during follow-up*			
	n	No diabetes (n = 7961)	Diabetes (n = 708)	p	n	No diabetes (n = 6455)	Diabetes (n = 506)	p
Age, mean (SD)	8669	66.46 (9.70)	69.38 (9.16)	<0.001	6961	66.02 (9.53)	65.51 (8.59)	<0.001
Sex, n (%)	8669			<0.001	6961			<0.001
Male		3522 (44.2%)	379 (53.5%)			2791 (43.2%)	262 (51.8%)	
Female		4439 (55.8%)	329 (46.5%)			3664 (56.8%)	244 (48.2%)	
Age left full-time education, n (%)	8468			<0.001	6809			<0.001
≤14 years		1641 (21.1%)	210 (30.6%)			1222 (19.3%)	107 (21.8%)	
15-16 years		4085 (52.5%)	349 (50.8%)			3283 (52.0%)	302 (61.6%)	
17-18 years		1009 (13.0%)	55 (8.0%)			870 (13.8%)	45 (9.2%)	
≥19 years		1046 (13.4%)	73 (10.6%)			944 (14.9%)	36 (7.3%)	
Social class, n (%)	8508			<0.001	6846			<0.001
Managerial and professional		2444 (31.2%)	194 (28.4%)			2067 (32.6%)	133 (26.7%)	
Intermediate		1979 (25.3%)	131 (19.2%)			1662 (26.2%)	104 (20.9%)	
Routine and manual		3403 (43.5%)	357 (52.3%)			2619 (41.3%)	261 (52.4%)	
Health literacy, n (%)	8293			<0.001	6736			<0.001
Adequate		5172 (67.7%)	376 (57.8%)			4351 (69.7%)	300 (61.2%)	
Limited		2471 (32.3%)	274 (42.2%)			1895 (30.3%)	190 (38.8%)	
Cognitive ability, mean (SD)	8335	0.03 (1.00)	-0.36 (0.97)	<0.001	6746	0.10 (0.98)	-0.04 (0.89)	<0.001
BMI, mean (SD)	7179	27.71 (4.79)	30.45 (5.37)	<0.001	5997	27.46 (4.64)	31.21 (5.28)	<0.001
Current smoker, n (%)	8622			0.377	6929			<0.001
Yes		1216 (15.4%)	99 (14.1%)			934 (14.5%)	105 (20.8%)	
No		6704 (84.6%)	603 (85.9%)			5490 (85.5%)	400 (79.2%)	
Alcohol, n (%)	7577			<0.001	6239			<0.001
Never		723 (10.3%)	112 (19.3%)			565 (9.7%)	49 (11.2%)	
Rarely		1076 (15.4%)	124 (21.3%)			863 (14.9%)	90 (20.6%)	

For peer review only

For peer review only - http://bmjopen.bmj.com/site/about/guidelines.xhtml

At least once a month		827 (11.8%)	85 (14.6%)			669 (11.5%)	70 (16.1%)	
At least once a week		2662 (38.1%)	171 (29.4%)			2255 (38.9%)	149 (34.2%)	
Daily/almost daily		1708 (24.4%)	89 (15.3%)			1451 (25.0%)	78 (17.9%)	
Physical activity, n (%)	8665			< 0.001	6958			<0.001
Vigorous activity		2236 (28.1%)	108 (15.2%)			1938 (30.0%)	116 (22.9%)	
Moderate activity		3888 (48.9%)	305 (43.1%)			3194 (49.5%)	233 (46.0%)	
Inactive		1833 (23.0%)	295 (41.7%)			1320 (20.5%)	157 (31.0%)	
Number of cardiovascular comorbidities, mean (SD)	8669	0.67 (0.91)	1.28 (1.13)	<0.001	6961	0.64 (0.88)	0.89 (1.04)	<0.001

BMI, body mass index.

*Diabetes reported at follow-up comparisons are based on a sub-sample of participants who did not self-report diabetes at wave 2 and with at least one wave of follow-up.

For peer review only - http://bmjopen.bmj.com/site/about/guidelines.xhtml

Table 2 Spearman rank-order correlations between covariates (n=6,463 to 8,660)

	Age	Sex	Education	Social class	Health literacy	Cognitive ability	BMI	Smoking	Alcohol	Physical activity	CV comorbid
Age											
Sex	-0.03**										
Education	-0.41***	0.02									
Social class	0.08***	-0.09***	-0.41***								
Health literacy	-0.16***	0.01	0.23***	-0.18***							
Cognitive ability	-0.47***	-0.09***	0.39***	-0.27***	0.31***						
BMI	-0.07***	0.02	-0.06***	0.08***	-0.04**	-0.01					
Smoking	-0.13***	0.01	-0.05***	0.12***	-0.04***	-0.02	-0.09***				
Alcohol	-0.11***	0.21***	0.22***	-0.20***	0.09***	0.16***	-0.11***	-0.04***			
Physical activity	-0.26***	0.10***	0.23***	-0.15***	0.14***	0.26***	-0.11***	-0.09***	0.18***		
CV comorbid	0.18***	0.00	-0.11***	0.05***	-0.06***	-0.11***	0.14***	-0.03*	-0.08***	-0.14***	

*p<.05, **p<.01, ***p<.001

BMI, body mass index; CV comorbid, number of cardiovascular comorbidities.

Sex is coded 0 for female, 1 for male; Education is age of leaving full-time education and is coded 1 for age 14 years or less, 2 for age 15-16 years, 3 for age 17-18 years, and 4 for 19 years or older; Social class is coded 1 for managerial and professional, 2 for intermediate, and 3 for routine and manual; Health literacy is coded 0 for limited and 1 for adequate; Smoking is coded 0 for current non-smoker and 1 for current smoker; Alcohol is the frequency of alcohol consumed in the last 12 months and is coded 0 for never, 1 for rarely, 2 for at least once a month, 3 for at least once a week, 4 for daily/almost daily; Physical activity is coded 0 for inactive, 1 for moderate activity at least once per week, 2 for vigorous activity at least once per week; CV comorbid is the number of cardiovascular comorbidities self-reported from hypertension, angina, heart attack, heart failure, heart murmur, abnormal heart rhythm, stroke, and high cholesterol.

For peer review only - http://bmjopen.bmj.com/site/about/guidelines.xhtml

Table 3 Odds ratios (95% CIs) from logistic regression models of the association between functional health literacy and cognitive ability with self-reported diabetes at wave 2

	Model 1: Health literacy	Model 2: Cognitive ability	Model 3: Health literacy and cognitive ability	Model 4: +BMI and health behaviours	Model 5: +CV comorbidities	Model 6: +Education and social class	Model 7: Fully-adjusted
Adequate health literacy	0.71*** (0.61, 0.84)	-	0.82* (0.69, 0.98)	0.97 (0.78, 1.21)	0.85 (0.72, 1.02)	0.84 (0.70, 1.01)	0.98 (0.78, 1.23)
Cognitive ability	-	0.73*** (0.67, 0.80)	0.78*** (0.70, 0.86)	0.90 (0.80, 1.02)	0.78*** (0.71, 0.87)	0.78*** (0.71, 0.87)	0.87 (0.76, 1.00)

*p<.05, **p<.01, ***p<.001

BMI, body mass index; CV, cardiovascular.

All models adjusted for age, age-squared, and sex. Model 1 n=8,293, Model 2 n=8,335, Model 3 n=8,185. Model 4 (n=6,302) adjusted for body mass index, frequency of alcohol consumption in the past 12 months, and physical activity. Model 5 (n=8,185) adjusted for number of cardiovascular comorbidities reported, and a squared term for number of cardiovascular comorbidities reported. Model 6 (n=7,861) adjusted for age left full-time education, and occupational social class. Model 7 (n=6,086) adjusted for all covariates.

For peer review only

Table 4 Hazard ratios (95% CIs) from Cox regression models of the association between functional health literacy and cognitive ability with self-reporting diabetes during follow-up

	Model 1: Health literacy	Model 2: Cognitive ability	Model 3: Health literacy and cognitive ability	Model 4: +BMI and health behaviours	Model 5: +CV comorbidities	Model 6: +Education and social class	Model 7: Fully-adjusted
Adequate health literacy	0.64*** (0.53, 0.77)	-	0.72*** (0.59, 0.87)	0.79* (0.64, 0.99)	0.73** (0.60, 0.88)	0.79* (0.65, 0.97)	0.85 (0.68, 1.06)
Cognitive ability	-	0.77*** (0.69, 0.85)	0.79*** (0.71, 0.88)	0.85* (0.74, 0.96)	0.80*** (0.71, 0.89)	0.84** (0.75, 0.95)	0.88 0.77, 1.01)

*$p<.05$, **$p<.01$, ***$p<.001$

BMI, body mass index, CV, cardiovascular.

All models adjusted for age and sex. Models 1 (n=6,736) had 490 diabetes events, Model 2 (n=6,746) had 491 diabetes events, Model 3 (n=6,654) had 484 diabetes events. Model 4 (n=5,357; 377 diabetes events) adjusted for body mass index, frequency of alcohol consumption in the past 12 months, and physical activity. Model 5 (n=6,654; 484 diabetes events) adjusted for number of cardiovascular comorbidities reported. Model 6 (n=6,409; 492 diabetes events) adjusted for age left full-time education, and occupational social class. Model 7 (n=5,186, 360 diabetes events) adjusted for all covariates.

For peer review only

Supplementary material for:

The association of functional health literacy and cognitive ability with self-reported diabetes in the English Longitudinal Study of Ageing: A prospective cohort study

For peer review only

For peer review only - http://bmjopen.bmj.com/site/about/guidelines.xhtml

Supplementary Table S1 Odds ratios (95% CI) from logistic regression models of the association between functional health literacy and cognitive ability with self-reported diabetes at wave 2

	Model 1: Health literacy n=8,293	Model 2: Cognitive ability n=8,335	Model 3: Health literacy and cognitive ability n=8,185	Model 4: +BMI and health behaviours n=6,302	Model 5: +CV comorbidities n=8,185	Model 6: +Education and social class n=7,861	Model 7: Fully-adjusted n=6,086
Health literacy							
Limited	Reference	-	Reference	Reference	Reference	Reference	Reference
Adequate	0.71***		0.82*	0.97	0.85	0.84	0.98
	(0.61, 0.84)		(0.69, 0.98)	(0.78, 1.21)	(0.72, 1.02)	(0.70, 1.01)	(0.78, 1.23)
Cognitive ability	-	0.73***	0.78***	0.90	0.78***	0.78***	0.87
		(0.67, 0.80)	(0.70, 0.86)	(0.80, 1.02)	(0.71, 0.87)	(0.71, 0.87)	(0.76, 1.00)
Age	1.04***	1.03***	1.03***	1.04***	1.02***	1.03***	1.03***
	(1.03, 1.05)	(1.02, 1.04)	(1.02, 1.04)	(1.03, 1.06)	(1.01, 1.03)	(1.02, 1.04)	(1.02, 1.05)
Age^2	0.998***	0.998***	0.998***	0.998**	0.998***	0.998***	0.999
	(0.997, 0.999)	(0.997, 0.998)	(0.997, 0.999)	(0.997, 0.999)	(0.997, 0.999)	(0.997, 0.999)	(0.998, 1.000)
Sex							
Female	Reference	Reference	Reference	Reference	Reference	Reference	Reference
Male	1.50***	1.41***	1.43	2.16***	1.45***	1.44***	2.09***
	(1.28, 1.77)	(1.20, 1.66)	(1.22, 1.69)	(1.75, 2.68)	(1.23, 1.71)	(1.22, 1.71)	(1.67, 2.62)
BMI				1.10***			1.09***
				(1.08, 1.12)			(1.07, 1.11)
Current smoking							
Non-smoker				Reference			Reference
Smoker				0.91			0.93
				(0.66, 1.23)			(0.66, 1.27)
Alcohol consumption							
Daily/almost daily				Reference			Reference
At least once per week				1.21			1.24
				(0.90, 1.65)			(0.91, 1.70)
At least once per month				1.78**			1.77**
				(1.24, 2.56)			(1.21, 2.57)
Rarely				1.95***			1.95***
				(1.38, 2.76)			(1.36, 2.79)
Never				2.40***			2.12***
				(1.67, 3.44)			(1.45, 3.11)
Physical activity							
Inactive				Reference			Reference

For peer review only

For peer review only - http://bmjopen.bmj.com/site/about/guidelines.xhtml

Moderate activity	0.65*** (0.51, 0.83)			0.68** (0.53, 0.87)
Vigorous activity	0.50*** (0.37, 0.68)			0.56*** (0.41, 0.76)
Number of CV comorbidities		2.08*** (1.84, 2.36)		1.98*** (1.70, 2.32)
Number of CV comorbidities^2		0.88*** (0.84, 0.93)		0.88*** (0.82, 0.93)
Age left full-time education				
≤14 years			Reference	Reference
15-16 years			1.06 (0.84, 1.34)	1.16 (0.87, 1.56)
17-18 years			0.81 (0.56, 1.14)	0.98 (0.63, 1.50)
≥19 years			1.06 (0.74, 1.50)	1.32 (0.85, 2.05)
Social class				
Managerial and professional			Reference	Reference
Intermediate			0.79 (0.61, 1.02)	0.79 (0.58, 1.07)
Routine and manual			1.08 (0.87, 1.35)	1.01 (0.77, 1.32)

*p<.05, **p<.01, ***p<.001

Age^2, age squared; BMI, body mass index; CV, cardiovascular; number of CV cormorbidities^2, number of cardiovascular comorbidities squared.

For peer review only

Supplementary Table S2 Hazard ratios (95% CI) from Cox regression models of the association between functional health literacy and cognitive ability with self-reporting diabetes during follow-up

	Model 1: Health literacy n=6,736 Events=490	Model 2: Cognitive ability n=6,746 Events=491	Model 3: Health literacy and cognitive ability n=6,654 Events=484	Model 4: +BMI health behaviours n=5,357 Events=377	Model 5: +CV comorbidities n=6654 Events=484	Model 6: +Education and social class n=6409 Events=462	Model 7: Fully-adjusted n=5,186 Events=360
Health literacy							
Limited	Reference	-	Reference	Reference	Reference	Reference	Reference
Adequate	0.64*** (0.53, 0.77)		0.72*** (0.59, 0.87)	0.79* (0.64, 0.99)	0.73** (0.60, 0.88)	0.79* (0.65, 0.97)	0.85 (0.68, 1.06)
Cognitive ability	-	0.77*** (0.69, 0.85)	0.79*** (0.71, 0.88)	0.85* (0.74, 0.96)	0.80*** (0.71, 0.89)	0.84** (0.75, 0.95)	0.88 (0.77, 1.01)
Age	1.01 (1.00, 1.02)	1.00 (0.99, 1.01)	1.00 (0.98, 1.01)	1.01 (1.00, 1.02)	0.99 (0.98, 1.00)	1.00 (0.98, 1.01)	1.01 (0.99, 1.02)
Sex							
Female	Reference	Reference	Reference	Reference	Reference	Reference	Reference
Male	1.43*** (1.20, 1.71)	1.39*** (1.16, 1.66)	1.38*** (1.15, 1.65)	1.84*** (1.49, 2.29)	1.38*** (1.16, 1.66)	1.39*** (1.15, 1.68)	1.82*** (1.45, 2.28)
BMI				1.12*** (1.10, 1.14)			1.12*** (1.10, 1.13)
Current smoking							
Non-smoker				Reference			Reference
Smoker				1.77*** (1.35, 2.31)			1.69*** (1.28, 2.22)
Alcohol consumption							
Daily/almost daily				Reference			Reference
At least once per week				1.11 (0.83, 1.49)			1.01 (0.75, 1.37)
At least once per month				1.53* (1.07, 2.19)			1.40 (0.97, 2.01)
Rarely				1.78*** (1.27, 2.50)			1.53* (1.08, 2.17)
Never				1.42 (0.95, 2.11)			1.15 (0.76, 1.73)
Physical activity							
Inactive				Reference			Reference
Moderate activity				0.78			0.79

For peer review only - http://bmjopen.bmj.com/site/about/guidelines.xhtml

	(0.61, 1.01)			(0.61, 1.03)
Vigorous activity	0.72* (0.54, 0.98)			0.76 (0.56, 1.04)
Number of CV comorbidities		1.34*** (1.22, 1.46)		1.17** (1.05, 1.30)
Age left full-time education				
≤14 years			Reference	Reference
15-16 years			0.93 (0.71, 1.22)	1.00 (0.74, 1.36)
17-18 years			0.61* (0.41, 0.91)	0.73 (0.47, 1.15)
≥19 years			0.44*** (0.28, 0.68)	0.58* (0.35, 0.96)
Social class				
Managerial and professional			Reference	Reference
Intermediate			0.81 (0.62, 1.07)	0.91 (0.66, 1.24)
Routine and manual			1.17 (0.93, 1.49)	1.17 (0.89, 1.53)

*p<.05, **p<.01, ***p<.001

BMI, body mass index; CV, cardiovascular.

For peer review only

For peer review only - http://bmjopen.bmj.com/site/about/guidelines.xhtml

Supplementary Table S3 Odds ratios (95% CI) for the association between functional health literacy and cognitive ability with cross-sectional diabetes status at wave 2 in a sub-sample of 6,086 participants with data on all variables of interest

	Model 1: Health literacy	Model 2: Cognitive ability	Model 3: Health literacy and cognitive ability	Model 4: +BMI and health behaviours	Model 5: +CV comorbidities	Model 6: +Education and social class	Model 7: Fully-adjusted
Health literacy							
Limited	Reference	-	Reference	Reference	Reference	Reference	Reference
Adequate	0.79* (0.64, 0.97)		0.88 (0.71, 1.10)	0.96 (0.77, 1.20)	0.92 (0.74, 1.15)	0.90 (0.72, 1.12)	0.98 (0.78, 1.23)
Cognitive ability	-	0.78*** (0.69, 0.88)	0.79*** (0.70, 0.90)	0.88 (0.77, 1.00)	0.80*** (0.70, 0.91)	0.82** (0.72, 0.93)	0.88 (0.77, 1.00)
Age	1.04*** (1.03, 1.06)	1.03*** (1.02, 1.05)	1.03*** (1.02, 1.05)	1.04*** (1.02, 1.05)	1.02** (1.01, to 1.04)	1.04*** (1.02, 1.05)	1.03*** (1.02, 1.05)
Age^2	0.999* (0.997, 1.000)	0.999** (0.997, 1.000)	0.999** (0.997, 1.000)	0.998** (0.997, 1.000)	0.999 (0.998, 1.000)	0.999* (0.997, 1.000)	0.999 (0.998, 1.000)
Sex							
Female	Reference	Reference	Reference	Reference	Reference	Reference	Reference
Male	1.66*** (1.36, 2.03)	1.58*** (1.29, 1.93)	1.58*** (1.29, 1.94)	2.17*** (1.74, 2.70)	1.63*** (1.33, 2.00)	1.56 (1.27, 1.92)	2.09*** (1.67, 2.62)
BMI				1.10*** (1.08, 1.12)			1.09*** (1.07, 1.11)
Current smoking							
Non-smoker				Reference			Reference
Smoker				0.89 (0.64, 1.22)			0.93 (0.66, 1.27)
Alcohol consumption							
Daily/almost daily				Reference			Reference
At least once per week				1.21 (0.90, 1.66)			1.24 (0.91, 1.70)
At least once per month				1.76** (1.21, 2.54)			1.77** (1.21, 2.57)
Rarely				2.01*** (1.42, 2.87)			1.95*** (1.36, 2.79)
Never				2.24*** (1.55, 3.26)			2.12*** (1.45, 3.11)
Physical activity							
Inactive				Reference			Reference
Moderate activity				0.65***			0.68**

For peer review only - http://bmjopen.bmj.com/site/about/guidelines.xhtml
For peer review only

	(0.51, 0.82)			(0.53, 0.87)
Vigorous activity	0.51***			0.56***
	(0.37, 0.69)			(0.41, 0.76)
Number of CV comorbidities		2.22***		1.98***
		(1.91, 2.59)		(1.70, 2.32)
Number of CV comorbidities^2		0.87***		0.88***
		(0.81, 0.92)		(0.82, 0.93)
Education				
≤14 years			Reference	Reference
15-16 years			1.07	1.17
			(0.81, 1.42)	(0.87, 1.56)
17-18 years			0.78	0.98
			(0.51, 1.18)	(0.64, 1.50)
≥19 years			0.94	1.32
			(0.62, 1.43)	(0.85, 2.05)
Social class				
Managerial and professional			Reference	Reference
Intermediate			0.79	0.79
			(0.59, 1.07)	(0.58, 1.07)
Routine and manual			1.09	1.01
			(0.84, 1.42)	(0.77, 1.32)

*p<.05, **p<.01, ***p<.001

Age^2, age squared; BMI, body mass index; CV, cardiovascular; number of CV cormorbidities^2, number of cardiovascular comorbidities squared.

For peer review only

For peer review only - http://bmjopen.bmj.com/site/about/guidelines.xhtml

Supplementary Table S4 Hazard ratios (95% CI) from Cox regression models of the association between functional health literacy and cognitive ability with self-reporting diabetes during follow-up. Models are run on a sub-sample of 5,186 (360 with diabetes) participants with data on all variables of interest

	Model 1: Health literacy	Model 2: Cognitive ability	Model 3: Health literacy and cognitive ability	Model 4: +BMI and health behaviours	Model 5: +CV comorbidities	Model 6: +Education and social class	Model 7: Fully-adjusted
Health literacy							
Limited	Reference	-	Reference	Reference	Reference	Reference	Reference
Adequate	0.64*** (0.52, 0.80)		0.73** (0.58, 0.91)	0.80 (0.64, 1.01)	0.74** (0.59, 0.93)	0.79* (0.63, 0.98)	0.85 (0.68, 1.06)
Cognitive ability	-	0.72*** (0.63, 0.82)	0.76*** (0.66, 0.86)	0.84** (0.73, 0.96)	0.76*** (0.67, 0.87)	0.83** (0.72, 0.95)	0.88 (0.77, 1.01)
Age	1.01 (0.997, 1.02)	1.00 (0.98, 1.01)	1.00 (0.98, 1.01)	1.01 (0.997, 1.03)	0.99 (0.98, 1.01)	1.00 (0.98, 1.01)	1.01 (0.99, 1.02)
Sex							
Female	Reference	Reference	Reference	Reference	Reference	Reference	Reference
Male	1.47*** (1.20, 1.81)	1.38** (1.12, 1.70)	1.40** (1.13, 1.72)	1.82*** (1.46, 2.27)	1.40** (1.14, 1.73)	1.42** (1.15, 1.76)	1.82*** (1.45, 2.28)
BMI				1.12*** (1.10, 1.14)			1.12*** (1.10, 1.13)
Current smoking							
Non-smoker				Reference			Reference
Smoker				1.79*** (1.36, 2.34)			1.69*** (1.28, 2.22)
Alcohol consumption							
Daily/almost daily				Reference			Reference
At least once per week				1.10 (0.80, 1.46)			1.01 (0.75, 1.36)
At least once per month				1.49* (1.03, 2.14)			1.40 (0.97, 2.01)
Rarely				1.70** (1.20, 2.40)			1.53* (1.08, 2.17)
Never				1.30 (0.86, 1.96)			1.15 (0.76, 1.73)
Physical activity							
Inactive				Reference			Reference
Moderate activity				0.76* (0.59, 0.99)			0.79 (0.61, 1.03)
Vigorous activity				0.71*			0.76

For peer review only - http://bmjopen.bmj.com/site/about/guidelines.xhtml

	(0.52, 0.96)			(0.56, 1.04)
Number of CV comorbidities		1.30*** (1.17, 1.45)		1.17** (1.05, 1.30)
Education				
≤14 years			Reference	Reference
15-16 years			0.90 (0.67, 1.23)	1.00 (0.74, 1.36)
17-18 years			0.56* (0.36, 0.88)	0.73 (0.47, 1.15)
≤19 years			0.44** (0.27, 0.73)	0.58* (0.35, 0.96)
Social class				
Managerial and professional			Reference	Reference
Intermediate			0.86 (0.63, 1.17)	0.91 (0.66, 1.24)
Routine and manual			1.24 (0.95, 1.63)	1.16 (0.89, 1.53)

*p<.05, **p<.01, ***p<.001

BMI, body mass index; CV, cardiovascular.

For peer review only

For peer review only - http://bmjopen.bmj.com/site/about/guidelines.xhtml

Supplementary Table S5 Odds ratios (95% CI) from logistic regression models of the association between functional health literacy and cognitive ability with self-reported diabetes at wave 2. Models are run on a sub-sample of participants with HbA_{1c} levels recorded wave 2, removing participants with suspected undiagnosed diabetes (n=5,671; 399 with diabetes)

	Model 1: Health literacy n= 5533	Model 2: Cognitive ability n=5534	Model 3: Health literacy and cognitive ability n=5470	Model 4: +BMI and health behaviours n=4845	Model 5: +CV comorbidities n=5470	Model 6: +Education and social class n=5271	Model 7: Fully-adjusted n=4674
Health literacy							
Limited	Reference	-	Reference	Reference	Reference	Reference	Reference
Adequate	0.91 (0.73, 1.14)		1.04 (0.82, 1.32)	1.17 (0.90, 1.53)	1.07 (0.84, 1.36)	1.04 (0.82, 1.33)	1.14 (0.87, 1.51)
Cognitive ability	-	0.78*** (0.69, 0.88)	0.78*** (0.69, 0.89)	0.88 (0.76, 1.03)	0.78*** (0.69, 0.89)	0.80** (0.69, 0.92)	0.85 (0.73, 1.01)
Age	1.04*** (1.03, 1.05)	1.03*** (1.02, 1.05)	1.03*** (1.01, 1.04)	1.04*** (1.02, 1.05)	1.02* (1.00, 1.03)	1.03*** (1.01, 1.04)	1.03** (1.01, 1.05)
Age^2	0.998*** (0.996, 0.999)	0.997*** (0.996, 0.999)	0.998*** (0.996, 0.999)	0.998** (0.996, 0.999)	0.998** (0.997, 0.999)	0.998** (0.996, 0.999)	0.999 (0.997, 1.000)
Sex							
Female	Reference	Reference	Reference	Reference	Reference	Reference	Reference
Male	1.45*** (1.17, 1.79)	1.39** (1.13, 1.72)	1.38** (1.11, 1.71)	2.16*** (1.67, 2.79)	1.40** (1.13, 1.74)	1.38** (1.10, 1.72)	2.09*** (1.60, 2.74)
BMI				1.11*** (1.09, 1.14)			1.10*** (1.07, 1.12)
Current smoking							
Non-smoker				Reference			Reference
Smoker				0.83 (0.56, 1.20)			0.86 (0.57, 1.26)
Alcohol consumption							
Daily/almost daily				Reference			Reference
At least once per week				1.33 (0.94, 1.92)			1.42 (0.98, 2.08)
At least once per month				1.87** (1.22, 2.87)			1.95** (1.25, 3.07)
Rarely				2.08*** (1.38, 3.16)			2.22*** (1.44, 3.44)
Never				2.23*** (1.43, 3.49)			1.85* (1.14, 3.00)

For peer review only

Physical activity				
Inactive	Reference			Reference
Moderate activity	0.65**			0.68*
	(0.49, 0.87)			(0.50, 0.92)
Vigorous activity	0.40***			0.43***
	(0.27, 0.57)			(0.29, 0.63)
Number of CV comorbidities		2.30***		2.08***
		(1.96, 2.70)		(1.73, 2.50)
Number of CV comorbidities^2		0.88***		0.90**
		(0.82, 0.94)		(0.82, 0.97)
Age left full-time education				
≤14 years			Reference	Reference
15-16 years			1.00	1.31
			(0.74, 1.37)	(0.92, 1.87)
17-18 years			0.63	0.94
			(0.39, 1.00)	(0.55, 1.58)
≥19 years			0.84	1.23
			(0.53, 1.32)	(0.72, 2.08)
Social class				
Managerial and professional			Reference	Reference
Intermediate			0.64**	0.62*
			(0.46, 0.89)	(0.42, 0.89)
Routine and manual			0.94	0.89
			(0.71, 1.24)	(0.65, 1.22)

*p<.05, **p<.01, ***p<.001

Age^2, age squared; BMI, body mass index; CV, cardiovascular; number of CV cormorbidities^2, number of cardiovascular comorbidities squared.

For peer review only

Supplementary Table S6 Hazard ratios (95% CI) from Cox regression models of the association between functional health literacy and cognitive ability with reporting diabetes during follow-up. Models are run on a sub-sample of participants with HbA_{1c} levels recorded at wave 4 and/or wave 6, removing participants with suspected undiagnosed diabetes (n=4,206; 212 with diabetes)

	Model 1: Health literacy n=3675 Events=203	Model 2: Cognitive ability n=3674 Events=205	Model 3: Health literacy and cognitive ability n=3641 Events=201	Model 4: +BMI health behaviours n=3184 Events=165	Model 5: +CV comorbidities n=3641 Events=201	Model 6: +Education and social class n=3530 Events=195	Model 7: Fully-adjusted n=3095 Events=159
Health literacy							
Limited	Reference	-	Reference	Reference	Reference	Reference	Reference
Adequate	0.50*** (0.38, 0.67)		0.58*** (0.43, 0.77)	0.63** (0.45, 0.88)	0.59*** (0.44, 0.79)	0.61*** (0.45, 0.82)	0.65* (0.46, 0.90)
Cognitive ability	-	0.71*** (0.60, 0.84)	0.78** (0.65, 0.94)	0.91 (0.74, 1.11)	0.78** (0.66, 0.94)	0.83* (0.68, 0.997)	0.93 (0.76, 1.16)
Age	1.02* (1.00, 1.03)	1.01 (0.99, 1.03)	1.01 (0.99, 1.03)	1.02* (1.00, 1.04)	1.00 (0.98, 1.02)	1.01 0.99, 1.03)	1.01 (0.99, 1.04)
Sex							
Female	Reference	Reference	Reference	Reference	Reference	Reference	Reference
Male	1.57** (1.19, 2.06)	1.50** (1.14, 1.98)	1.51** (1.14, 2.00)	2.22 *** (1.59, 3.10)	1.53** (1.15, 2.02)	1.50** (1.12, 2.00)	2.11*** (1.49, 2.97)
BMI				1.12*** (1.10, 1.15)			1.11*** (1.08, 1.14)
Current smoking							
Non-smoker				Reference			Reference
Smoker				2.12*** (1.43, 3.15)			2.04*** (1.36, 3.06)
Alcohol consumption							
Daily/almost daily				Reference			Reference
At least once per week				1.09 (0.70, 1.71)			1.00 (0.64, 1.57)
At least once per month				1.48 (0.86, 2.55)			1.34 (0.77, 2.34)
Rarely				2.10*** (1.27, 3.48)			1.78* (1.06, 2.98)
Never				1.54 (0.85, 2.80)			1.27 (0.69, 2.35)
Physical activity							
Inactive				Reference			Reference

For peer review only - http://bmjopen.bmj.com/site/about/guidelines.xhtml

Moderate activity	0.64* (0.43, 0.94)			0.65* (0.44, 0.96)
Vigorous activity	0.61* (0.39, 0.95)			0.65 (0.41, 1.02)
Number of CV comorbidities		1.43*** (1.25, 1.64)		1.22* (1.04, 1.44)
Age left full-time education				
≤14 years			Reference	Reference
15-16 years			1.01 (0.66, 1.54)	0.91 (0.57, 1.44)
17-18 years			0.71 (0.39, 1.29)	0.78 (0.41, 1.48)
≥19 years			0.52 (0.27, 1.02)	0.59 (0.28, 1.23)
Social class				
Managerial and professional			Reference	Reference
Intermediate			0.83 (0.54, 1.27)	0.84 (0.52, 1.35)
Routine and manual			1.22 (0.85, 1.74)	1.20 (0.80, 1.79)

*p<.05, **p<.01, ***p<.001

BMI, body mass index; CV, cardiovascular.

For peer review only

For peer review only - http://bmjopen.bmj.com/site/about/guidelines.xhtml

STROBE Statement—checklist of items that should be included in reports of observational studies

	Item No	Recommendation	
**Title and abstract**	1	(*a*) Indicate the study's design with a commonly used term in the title or the abstract	Page 1
		(*b*) Provide in the abstract an informative and balanced summary of what was done and what was found	Page 2
**Introduction**			
Background/rationale	2	Explain the scientific background and rationale for the investigation being reported	Pages 5-7
Objectives	3	State specific objectives, including any prespecified hypotheses	Page 7
**Methods**			
Study design	4	Present key elements of study design early in the paper	Pages 6-8
Setting	5	Describe the setting, locations, and relevant dates, including periods of recruitment, exposure, follow-up, and data collection	Pages 7-8
Participants	6	(*a*) *Cohort study*—Give the eligibility criteria, and the sources and methods of selection of participants. Describe methods of follow-up<br>*Case-control study*—Give the eligibility criteria, and the sources and methods of case ascertainment and control selection. Give the rationale for the choice of cases and controls<br>*Cross-sectional study*—Give the eligibility criteria, and the sources and methods of selection of participants	Pages 7
		(*b*) *Cohort study*—For matched studies, give matching criteria and number of exposed and unexposed<br>*Case-control study*—For matched studies, give matching criteria and the number of controls per case	NA
Variables	7	Clearly define all outcomes, exposures, predictors, potential confounders, and effect modifiers. Give diagnostic criteria, if applicable	Pages 8-12
Data sources/ measurement	8*	For each variable of interest, give sources of data and details of methods of assessment (measurement). Describe comparability of assessment methods if there is more than one group	Pages 8-12
Bias	9	Describe any efforts to address potential sources of bias	Pages 18-19
Study size	10	Explain how the study size was arrived at	Pages 8, 14, 16
Quantitative variables	11	Explain how quantitative variables were handled in the analyses. If applicable, describe which groupings were chosen and why	Pages 12-13
Statistical methods	12	(*a*) Describe all statistical methods, including those used to control for confounding	Pages 12-13
		(*b*) Describe any methods used to examine subgroups and interactions	NA
		(*c*) Explain how missing data were addressed	Pages 18
		(*d*) *Cohort study*—If applicable, explain how loss to follow-up was addressed<br>*Case-control study*—If applicable, explain how matching of cases and controls was addressed	Pages 13

For peer review only

		Cross-sectional study—If applicable, describe analytical methods taking account of sampling strategy	
		(*e*) Describe any sensitivity analyses	Pages 18-19
**Results**			
Participants	13*	(a) Report numbers of individuals at each stage of study—eg numbers potentially eligible, examined for eligibility, confirmed eligible, included in the study, completing follow-up, and analysed	Pages 8, 14, 16
		(b) Give reasons for non-participation at each stage	Pages 8, 14, 16
		(c) Consider use of a flow diagram	NA
Descriptive data	14*	(a) Give characteristics of study participants (eg demographic, clinical, social) and information on exposures and potential confounders	Pages 14, 16, Table 1
		(b) Indicate number of participants with missing data for each variable of interest	Table 1
		(c) *Cohort study*—Summarise follow-up time (eg, average and total amount)	Pages 16
Outcome data	15*	*Cohort study*—Report numbers of outcome events or summary measures over time	Tables 3 and 4
		Case-control study—Report numbers in each exposure category, or summary measures of exposure	
		Cross-sectional study—Report numbers of outcome events or summary measures	Table 3 and 4
Main results	16	(*a*) Give unadjusted estimates and, if applicable, confounder-adjusted estimates and their precision (eg, 95% confidence interval). Make clear which confounders were adjusted for and why they were included	Tables 1-4
		(*b*) Report category boundaries when continuous variables were categorized	
		(*c*) If relevant, consider translating estimates of relative risk into absolute risk for a meaningful time period	NA
Other analyses	17	Report other analyses done—eg analyses of subgroups and interactions, and sensitivity analyses	Pages 18-19
**Discussion**			
Key results	18	Summarise key results with reference to study objectives	Page 20
Limitations	19	Discuss limitations of the study, taking into account sources of potential bias or imprecision. Discuss both direction and magnitude of any potential bias	Pages 22-24
Interpretation	20	Give a cautious overall interpretation of results considering objectives, limitations, multiplicity of analyses, results from similar studies, and other relevant evidence	Pages 24-25
Generalisability	21	Discuss the generalisability (external validity) of the study results	Pages 24-25
**Other information**			
Funding	22	Give the source of funding and the role of the funders for the present study and, if applicable, for the original study on which the present article is based	Page 26

*Give information separately for cases and controls in case-control studies and, if applicable, for exposed and unexposed groups in cohort and cross-sectional studies.

For peer review only

Note: An Explanation and Elaboration article discusses each checklist item and gives methodological background and published examples of transparent reporting. The STROBE checklist is best used in conjunction with this article (freely available on the Web sites of PLoS Medicine at http://www.plosmedicine.org/, Annals of Internal Medicine at http://www.annals.org/, and Epidemiology at http://www.epidem.com/). Information on the STROBE Initiative is available at www.strobe-statement.org.

For peer review only

For peer review only - http://bmjopen.bmj.com/site/about/guidelines.xhtml
